# LncRNA LINC01018/miR‐942‐5p/KNG1 axis regulates the malignant development of glioma in vitro and in vivo

**DOI:** 10.1111/cns.14053

**Published:** 2022-12-22

**Authors:** Jinfang Xu, Jianli Wang, Mingfei Zhao, Chenguang Li, Shen Hong, Jianmin Zhang

**Affiliations:** ^1^ Department of Neurosurgery The Second Affiliated Hospital Zhejiang University School of Medicine Hangzhou Zhejiang China

**Keywords:** glioma, KNG1, LINC01018, miR‐942‐5p

## Abstract

**Aims:**

Since the inhibitory effect of KNG1 on glioma has been proved, this study further explores the regulation of the lncRNA/miRNA axis on KNG1 in glioma.

**Methods:**

The miRNAs that target KNG1 and the lncRNA that targets miR‐942‐5p were predicted by bioinformatics analysis and verified by experiments. The correlations between miR‐942‐5p and the survival of patients and between KNG1 and miR‐942‐5p were analyzed. After transfection, cell migration, invasion, proliferation, and cell cycle were detected through wound healing, Transwell, colony formation, and flow cytometry assays. A mouse subcutaneous xenotransplanted tumor model was established. The expressions of miR‐942‐5p, KNG1, LINC01018, and related genes were evaluated by quantitative real‐time reverse transcription polymerase chain reaction (RT‐qPCR), Western blot, or immunohistochemistry.

**Results:**

MiR‐942‐5p targeted KNG1, and LINC01018 sponged miR‐942‐5p. The high survival rate of patients was related to low miR‐942‐5p level. MiR‐942‐5p was highly expressed, whereas KNG1 was lowly expressed in glioma. MiR‐942‐5p was negatively correlated with KNG1. Silent LINC01018 or KNG1 and miR‐942‐5p mimic enhanced the migration, invasion, and proliferation of glioma cells, and regulated the expressions of metastasis‐related and proliferation‐related genes. LINC01018 knockdown and miR‐942‐5p mimic promoted glioma tumor growth in mice. The levels of miR‐942‐5p and KNG1 were decreased by LINC01018 knockdown, and LINC01018 expression was suppressed by miR‐942‐5p mimic. MiR‐942‐5p inhibitor, KNG1, and LINC01018 had the opposite effect to miR‐942‐5p mimic.

**Conclusion:**

LINC01018/miR‐942‐5p/KNG1 pathway regulates the development of glioma cells in vitro and in vivo.

## Graphical abstract

Since the inhibitory effect of KNG1 on glioma had previously been proved, this study further explored the regulation of lncRNA/miRNA axis on KNG1 in glioma. In detail, the miRNAs that target KNG1 and the lncRNA that targets miR‐942‐5p were predicted by bioinformatics analysis and verified by experiments. The relationship between miR‐942‐5p and the survival of patients as well as the correlation between KNG1 and miR‐942‐5p were analyzed. After transfection, cell migration, invasion, proliferation, and cell cycle were detected through wound healing assay, Transwell assay, colony formation assay, and flow cytometry assay. Mice subcutaneous xenotransplanted tumor model was established. The expressions of miR‐942‐5p, KNG1, LINC01018, and related genes were evaluated by quantitative real‐time reverse transcription polymerase chain reaction (RT‐qPCR), western blot, or immunohistochemistry. And we found that MiR‐942‐5p targeted KNG1, and LINC01018 sponged miR‐942‐5p. High survival rate of patients was related to low miR‐942‐5p level. MiR‐942‐5p was highly expressed, whereas KNG1 was lowly expressed in glioma. MiR‐942‐5p was negatively correlated with KNG1. Silenced LINC01018 or KNG1 and miR‐942‐5p mimic enhanced the migration, invasion, and proliferation of glioma cells and regulated the expressions of metastasis‐related and proliferation‐related genes. shRNA of LINC01018 and miR‐942‐5p mimic promoted glioma tumor growth in mice. The levels of miR‐942‐5p and KNG1 were inhibited by shRNA of LINC01018, and LINC01018 expression was suppressed by miR‐942‐5p mimic. MiR‐942‐5p inhibitor, KNG1, and LINC01018 had the opposite effect to miR‐942‐5p mimic.

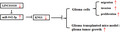

## 1 INTRODUCTION

Glioma, the most common malignant tumor of the central nervous system with high mortality, accounts for about 60% of all primary brain tumors worldwide.^1^, ^2^ Owing to its ability to rapidly proliferate, malignant glioma has a high potential of infiltrating surrounding normal tissues.^3^ Despite considerable advances in the treatment of glioma, such as surgery combined with radiotherapy or chemotherapy, therapeutic results remain unsatisfactory.^4^, ^5^ So far, the molecular mechanisms underlying the pathogenesis of glioma are still largely unknown, making it difficult to develop effective treatment methods for glioma.^6^, ^7^ Hence, it is necessary to figure out the potential mechanisms involved in the progression of glioma and formulate feasible therapeutic methods for glioma.

As a precursor protein of vasoactive kinin, *KNG1* is closely correlated with the progression of many diseases and cancers.^8^, ^9^, ^10^ Besides, *KNG1* has been identified as a biomarker for colorectal cancer, ovarian carcinoma, etc., with the ability to modulate the progression of different diseases.^8^, ^11^, ^12^ Recently, low expression of *KNG1* has been found in the serums of glioma patients.^13^ In addition, our previous research also proved that *KNG1* overexpression could inhibit proliferation and induce apoptosis in glioma cells.^13^ However, the deep mechanism of *KNG1* in glioma still awaits further discovery. Considering that microRNAs (miRNAs) could specifically target a certain mRNA to affect the pathogenesis of various diseases,^14^ we wondered whether *KNG1* could be regulated by some miRNAs and thereby further affects the progression of glioma.

MiRNAs, a kind of small noncoding RNA molecules, play critical roles in nearly all biological processes.^15^ Considerable evidence has indicated that abnormal levels of miRNAs, such as miR‐155, miR‐320a, miR‐130a, and miR‐93, are highly associated with the occurrence and development of glioma.^16^, ^17^, ^18^, ^19^, ^20^, ^21^ Moreover, it has been demonstrated that the effect of miRNAs in glioma can be realized by targeting some mRNAs. For example, miR‐181b‐5p regulates the chemosensitivity of glioma cells by targeting *Bcl‐2*,^22^ miR‐134‐5p modulates the capacities of glioma cells to proliferate and migrate through regulating *BTG2*,^23^ and *SND1* is targeted by miR‐361‐5p to suppress the metastasis of glioma.^24^ Nonetheless, whether the effect of *KNG1* on glioma is achieved via targeting a certain miRNA is yet to be investigated.

Additionally, it is widely recognized that the miRNA/mRNA axis is modulated by long noncoding RNAs (lncRNAs) since lncRNAs could competitively bind with miRNAs to further regulate the downstream signal transduction of miRNAs.^25^ For instance, *lncRNA CASC2* targets miR‐181a/*PTEN* axis to regulate the proliferation of glioma cells^26^; *lncRNA DLEU2*/miR‐186‐5p/*PDK3* axis promotes the development of giloma^27^; and *lncRNA SNHG5* enhances the proliferation of glioma cells by regulating miR‐205‐5p/*ZEB2* axis expression.^28^ Nevertheless, whether *KNG1* is also targeted by an lncRNA/miRNA axis to influence glioma remains unclear.

The purpose of this study was to seek the potential lncRNA and miRNA that regulate *KNG1* to affect the development of glioma. After performing a series of in vitro *and* in vivo experiments, we unraveled that *LINC01018*/miR‐942‐5p/*KNG1* axis regulated the malignant development of glioma cells, revealing a new regulatory pathway for glioma.

## 2 MATERIALS AND METHODS

### 2.1 Ethics statement

The use of clinical tissues was authorized by the Ethics Committee of the Second Affiliated Hospital Zhejiang University School of Medicine (Z20180708N), and all patients signed the written informed consent. Animal experiments were ratified by the Committee of Experimental Animals of the Second Affiliated Hospital Zhejiang University School of Medicine (Z20181021N). All experiments in this study were performed in the Second Affiliated Hospital Zhejiang University School of Medicine.

### 2.2 Tissue samples

Cancer lesions and adjacent normal samples (nonneoplastic normal brain tissues far from the tumor margins) were harvested from 52 glioma patients who underwent surgical excision at the Second Affiliated Hospital of Zhejiang University School of Medicine between March 2017 and March 2018. The patients (30 males and 22 females, 55 ± 6.3 years old) involved in this study were diagnosed with grade III or IV glioma according to WHO criteria.^29^ None of the patients received chemotherapy or radiotherapy prior to the surgery, and all brain tumor tissues were obtained at the primary resection.

### 2.3 Bioinformatics analysis

The target miRNAs of *KNG1* and the lncRNA‐targeting miR‐942‐5p were analyzed through TargetScan (http://www.targetscan.org/vert_72/), miRDB (http://mirdb.org/), miRWalk (http://mirwalk.umm.uni‐heidelberg.de/), and starBase (http://starbase.sysu.edu.cn/starbase2/). Among these databases, the prediction by starBase was based on the miRNA–mRNA interactions supported by Ago CLIP‐seq Data.^30^ Gene expression levels in glioma tissues and normal tissues were predicted using GEPIA2 (http://gepia2.cancer‐pku.cn/#index) based on the TCGA and GTEx databases. The binding sites of *KNG1* and *LINC01018* on miR‐942‐5p were predicted through Targetscan and starBase, which also presented the overall survival of miR‐942‐5p or miR‐455‐5p in brain lower‐grade glioma (LGG). The Brain LGG (TCGA, PanCancer Atlas) database on the cBioPortal website (https://www.cbioportal.org/) was used to assess the relationship between miR‐942‐5p copy number and histological grade, and the percentage of cases with new neoplasms in patients.

### 2.4 Cell culture

Normal human astrocytes (NHA; CRL‐1718) and human glioma cell lines (A172 [CRL‐1620], LN‐229 [CRL‐2611], and T98G [CRL‐1690]) were obtained from ATCC (Rockville, MD, USA). Human glioma cell lines U‐251 (1101HUM‐PUMC000058) and U‐251MG (4201HUM‐CCTCC00093) were obtained from China Center for Type Culture Collection (Beijing, China). NHA cells were cultured in RPMI‐1640 medium (C11875500BT; Gibco) containing 10% fetal bovine serum (FBS, 10437010, Gibco) and 1% penicillin–streptomycin (P/S; 15070063, Gibco). A172, LN‐229, T98G, U‐251MG, and U‐251 cells were cultivated in Dulbecco's Modified Eagle's Medium (DMEM, C11995500BT, Gibco) supplemented with 10% FBS and 1% P/S. All cells were cultured in a 37°C humid environment with 5% CO_2_.

### 2.5 Transfection

KNG1 overexpression plasmid, LINC01018 overexpression plasmid, and corresponding negative control (NC) were synthesized in Tiandz (Beijing, China). The whole sequences were ligated into the pcDNA3.1 vector (60908‐1440, Tiandz) before the construction of the plasmid. ShRNA for *LINC01018* (5’‐TCCCAAAGAGGTTACAATCATTA‐3′), shRNA NC (5’‐AATTCTCCGAACGTGTCACGT‐3′), siRNA for *KNG1* (siKNG1; target senquence: 5’‐CTGGAGATTGTCAAATTCAGTAT‐3′), siRNA NC (siNC; 5’‐UUUGUACUACACAAAAGUACUG‐3′), miR‐942‐5p mimic (5’‐UCUUCUCUGUUUUGGCCAUGUG‐3′), miR‐942‐5p inhibitor (5’‐CACAUGGCCAAAACAGAGAAGA‐3′), mimic control (5’‐UUCUCCGAACGUGUCACGUUU‐3′), and inhibitor control (5′‐ CAGUCCUUUUGUGUAGUACAA‐3′) were obtained from RIBOBIO (Guangzhou, China).

Before transfection, 1.0 × 10^6^ cells were inoculated into 6‐well plates with 2 ml of complete medium in each well. After the cell density reached 80%, 4 μg of plasmids, and 100 nmol/L siRNA, shRNA, mimic, or inhibitor were separately transfected into the cells using 3 μl of Lipofectamine 2000 (11668–019; Invitrogen). Then the cells were incubated at 37°C for 48 h.

### 2.6 Wound healing assay

About 3.5 × 10^5^ transfected cells were added to six‐well plates and cultured until reaching 100% confluence. Then, a vertical wound was created by a pipette tip in each well, and a medium without FBS was applied to further incubate the cells. At 0 h and 24 h after the wound creation, the images of the wounds were recorded using an optical microscope (eica, Solms, Germany), and the migration data were analyzed using the Image J 1.8.0 software.

### 2.7 Transwell assay

Transwell chambers precoated by Matrigel (354,234; Corning) were first inserted into a 24‐well plate. Then, 1 × 10^5^ transfected cells in 0.2 ml FBS‐free medium were added to the chambers, and meanwhile, 0.7 ml of medium with 10% FBS was added to the 24‐well plate. Following incubation for 24 h, the invasive cells were fixed by paraformaldehyde fixative (P0099; Beyotime) and stained by crystal violet (c805211; Macklin) at room temperature for 15 min. The invasive cells were photographed, the number of cells was counted using an optical microscope (×250), and the invasion data were analyzed by the Image J 1.8.0 software.

### 2.8 Colony formation assay

When transfection was completed, 1000 cells were cultured on a six‐well plate for 14 days for colony formation, with the medium being refreshed every 2 days. Then the cell colonies were fixed with paraformaldehyde fixative and stained by crystal violet for 15 min. After being washed three times with phosphate‐buffered saline (PBS), the number of visible colonies was counted and analyzed by the Image J 1.8.0 software.

### 2.9 Dual‐luciferase reporter assay

The *KNG1* wide‐type (wt) sequence (5′‐GGATAGAATTTAAATAGAGAAGA‐3′), and mutant (mut) sequence (5’‐GGATAGAATTTAAATTAGCGGCA‐3′), as well as *LINC01018* wt sequence (5’‐GGGAAAUCCAGAAACUCUAGAGAAGC‐3′) and mut sequence (5’‐GUGAAAUCCAGAGACUCUAGAGAAGC‐3′) were cloned into pmirGLO luciferase vectors (E1330; Promega). Then, 3.0 × 10^4^ LN‐2998 and T98G cells in 300 μl medium were added to each well of 48‐well plates and grown overnight. Then the vectors were transfected together with miR‐942‐5p mimic or its control into the cells for 48 h. After that, the cells were subjected to dual‐luciferase reporter assay (E1910; Promega), and the cell luciferase activity was evaluated by a GloMax fluorescence reader (Promega).

### 2.10 Flow cytometry

The cell cycle distribution in transfected cells was estimated using a Cell Cycle Detection Kit (KGA512; KeyGen). In brief, 2.0 × 10^5^ transfected cells were incubated with 70% ethanol (E111991; Aladdin) at 4°C for 16 h. After being washed with PBS twice, the cells were incubated with propidium iodide (PI) for 45 min in the dark. Last, the fluorescence was analyzed by a FACSCaliburTM flow cytometer (BD Biosciences), and the cell cycle distribution was analyzed using the FCS Express 3.0 software (Dickinson Biosciences).

### 2.11 Animals and subcutaneous xenograft

In this study, 80 male BALB/c nude mice (6‐week‐old, 20–22 g) were obtained from SLAC (Shanghai, China). All animals were fed in a specific pathogen‐free (SPF) environment under a 12‐h‐dark/12‐h‐light cycle and were divided into eight groups (*n* = 10): the NC group, the LINC01018 group, the mimic group, the LINC01018 + mimic group, the shRNA NC group, the shRNA LINC01018 group, the inhibitor group, and the shRNA LINC01018 + inhibitor group.

LN‐299 cells were transfected with NC, LINC01018 overexpression plasmids or miR‐942‐5p mimic, or co‐transfected with LINC01018 plasmids and miR‐942‐5p mimic. Then, mice in the NC group, the LINC01018 group, the mimic group, and the LINC01018 + mimic group were subcutaneously injected with the transfected LN‐299 cells (2 × 10^6^) in the right flank area. Meanwhile, T98G cells were transfected with shRNA NC, shRNA of LINC01018 or miR‐942‐5p inhibitor, or co‐transfected with shRNA of LINC01018 and miR‐942‐5p inhibitor. Then, mice in the shRNA NC, the shRNA LINC01018, the inhibitor, and the shRNA LINC01018 + inhibitor groups were subcutaneously injected with the transfected T98G cells (2 × 10^6^) in the right flank area.

Four weeks after the injection, all mice were anesthetized with 2% sodium pentobarbital (B005, Jiancheng) and sacrificed by cervical dislocation. Then, the tumor tissues were removed, weighed, photographed, and used in later experiments.

### 2.12 Quantitative real‐time reverse transcription polymerase chain reaction (RT‐qPCR)

MiRNAs in clinical samples, tumor tissues, and different cell lines were isolated using a miRNA extraction kit (TianGEN). Total RNAs in different cell lines were isolated using TRIzol reagent (15,596, Invitrogen). Then, the extracted RNA was reverse‐transcribed into cDNA with the help of a PrimeScript RT kit (RR037A; Takara). Finally, the gene expression in different samples was analyzed in Real‐Time PCR System (ABI 7500; Applied Biosystems) after the RT‐qPCR Master Mix (A15300; Thermo Scientific) was mixed with cDNA and gene primers (Table 1).

**TABLE 1:** q‐PCR primers

Target gene	Forward primers, 5′‐3′	Reverse primers, 5′‐3′
miR‐942‐5p miR‐455‐5p LINC01018	GTACTCACAGCCCCTCACAC TGCCTTTGGACTACATCGGT GTAAATGAGGCTCCTGGGA	CGGCAATTGCACTGGATACG TGTCGTGGAGTCGGCAATTG ATATTCTGTCTCCACCGCC
KNG1	TGCTCCAGGCTGCTACTAAGT	GGCTTCAGTTATGCGGTACAA
SNAIL1	TCGGAAGCCTAACTACAGCGA	AGATGAGCATTGGCAGCGAG
TWIST1 Vimentin ZEB1 N‐Cadherin E‐Cadherin ZO1 U6 GAPDH	GTCCGCAGTCTTACGAGGAG GACGCCATCAACACCGAGTT GATGATGAATGCGAGTCAGATGC TCAGGCGTCTGTAGAGGCTT CGAGAGCTACACGTTCACGG CAACATACAGTGACGCTTCACA CTCGCTTCGGCAGCACA GGAGCGAGATCCCTCCAAAAT	GCTTGAGGGTCTGAATCTTGCT CTTTGTCGTTGGTTAGCTGGT ACAGCAGTGTCTTGTTGTTGT ATGCACATCCTTCGATAAGACTG GGGTGTCGAGGGAAAAATAGG CACTATTGACGTTTCCCCACTC AACGCTTCACGAATTTGCGT GGCTGTTGTCATACTTCTCATGG

### 2.13 RNA pull‐down assay

This assay was performed as previously delineated.^31^ In brief, 50 nmol/L biotinylated miR‐942‐5p wt (WT‐bio‐miR‐942‐5p) and miR‐942‐5p mut (MUT‐bio‐miR‐942‐5p) were transfected into LN‐229 and T98G cells. After the cells were lysed and precoated with RNase‐free BSA (ST025, Beyotime) and yeast tRNA (T8630; Solarbio) at 4°C for 4 h, streptavidin magnetic beads (S3762; Chemical Company) were applied for incubation. Then, cells were washed with salt buffer four times. Finally, the bound RNA was purified, and LINC01018 enrichment was analyzed by RT‐qPCR analysis as described above.

### 2.14 RNA immunoprecipitation (RIP) assay

This assay was performed using a RIP kit (17–700) purchased from Millipore (MA, USA). Briefly,^31^ LN‐229 and T98G cells were collected and incubated with magnetic beads and anti‐Ago2 antibody (04‐642, Millipore) overnight, and Input and normal IgG (IG266; Abcam) were used as negative controls. Then, RNAs were extracted, and the relative enrichment of LINC01018 and miR‐942‐5p was determined by RT‐qPCR analysis as introduced in the section on *RT‐qPCR*.

### 2.15 Western blot assay

Total proteins in tumor tissues or different cell lines were extracted using RIPA lysis buffer (P0013B, Beyotime), and the protein concentration was evaluated using a BCA assay kit (23,250, Pierce, MA, USA). Then 25 μg of total protein was separated in SDS‐PAGE gels (P0052A, Beyotime), and further transferred onto the PVDF membranes (FFP32, Beyotime). Subsequently, the membranes were soaked in 5% skimmed milk for 2 h and further incubated with primary antibodies against KNG1 (1:3000, ab97761, 48 kD, Abcam, CA, USA), SNAIL1 (1:2000, ab53519, 29kD, Abcam), TWIST1 (1:1000, ab50887, 21kD, Abcam), Vimentin (1:5000, ab92547, 54kD, Abcam), ZEB1 (1:2000, ab180905, 124kD, Abcam), N‐Cadherin (1:2000, ab18203, 130kD, Abcam), E‐Cadherin (1:10000, ab40772, 97kD, Abcam), ZO1 (1:3000, ab96587, 187kD, Abcam), CDC25A (1:1000, ab989, 59kD, Abcam), cyclin D1 (1:1000, ab134175, 34kD, Abcam), CDKN2A (1:4000, ab201980, 17kD, Abcam), and GAPDH (1:1000, 36kD, ab8245, Abcam). The next day, the membranes were then incubated with Goat Anti‐Mouse (1:5000, ab205719, Abcam), Goat Anti‐Rabbit (1:5000, A0208, Beyotime), and Donkey Anti‐Goat (1:5000, ab205723, Abcam) secondary antibodies for 1 h. Finally, the protein signal in the membranes was detected under Image Lab system 3.0 (Bio‐Rad; Hercules) after the membranes were incubated with detection buffer (P0272, Beyotime) for 1 min.

### 2.16 Immunohistochemical analysis

The collected tumor tissue was fixed, embedded in paraffin (S25190; Yuanye), and cut into 4‐μm‐thick slices. Then the slices were soaked in antigen repair solution (P0081, Beyotime) for 10 min. After being blocked with 5% FBS for 1 h, the slices were incubated with anti‐KNG1 antibody (1:500, ab97761, Abcam) at 4°C overnight, followed by incubation with the corresponding secondary antibody (G‐21234, 1:500, Thermo Scientific) for 30 min. The CD31 antibody (1:500, ab9498, Abcam) was used to stain vascular endothelial cells, and then the microvessel density (MVD) was calculated. The slices or cells were treated with the DAB reagent (SFQ004; 4A Biotech) for 30 min and stained with hematoxylin (C0107, Beyotime) for 10 min. Last, images of the slices were observed and documented with a phase‐contrast optical microscope.

### 2.17 Statistical analysis

All data were analyzed by one‐way analysis of variance (ANOVA) followed by Tukey's post hoc test and independent *t* test in GraphPad 8.0. The normality of continuous variables was tested using the Shapiro‐Wilktest method. Pearson's correlation analysis was used to analyze the correlation between the expression of *KNG1* and that of miR‐942‐5p or miR‐455‐5p. The data were presented as mean ± standard deviation. *p* < 0.05 represented that the data were statistically significant.

## 3 RESULTS

### 3.1 *KNG1* was lowly expressed in glioma and negatively correlated to miR‐942‐5p which was highly expressed in glioma

**FIGURE 1:**
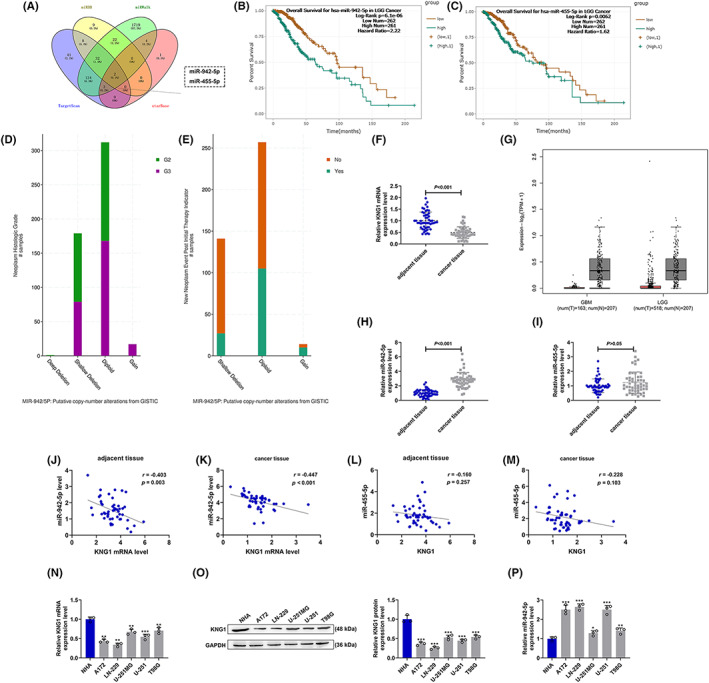
*KNG1* was lowly expressed in glioma and was negatively correlated to miR‐942‐5p which was highly expressed in glioma. (A) The miRNAs which might target *KNG1* were predicted through bioinformatics analysis (TargetScan, starBase, miRDB, and miRWalk). (B, C) The correlation between the expression levels of miR‐942‐5p and miR‐455‐5p with the overall survival of brain lower‐grade glioma (LGG) cancer patients was predicted through bioinformatics analysis and analyzed by Kaplan–Meier method (log‐rank test). (D, E) The correlation among miR‐942‐5p copy number, neoplasm histologic grade and new neoplasm event post initial therapy. (F) The expression of *KNG1* in clinical glioma tissues was analyzed by RT‐qPCR, with GAPDH serving as an internal control. (G) The expression of *KNG1* in glioma was predicted through bioinformatics analysis. (H, I) The expressions of miR‐492‐5p (H) and miR‐455‐5p (I) in clinical glioma tissues were analyzed by RT‐qPCR, with U6 serving as an internal control. (J–M) The correlation between *KNG1* and miR‐492‐5p (J, K) or miR‐455‐5p (L, M) in the normal and cancer samples from glioma patients was analyzed by Pearson's correlation analysis. (N–P) The expressions of *KNG1* (N, O) and miR‐492‐5p (P) in normal human astrocytes and human glioma cell lines were analyzed by RT‐qPCR or Western blot, with GAPDH or U6 serving as an internal control, respectively. (*p < 0.05, **p < 0.01, ***p < 0.001 vs. NHA).

Bioinformatics analysis was applied to analyze gene expression changes in glioma to determine the essential genes for glioma generation. MiR‐942‐5p and miR‐455‐5p might be the miRNAs that could target *KNG1* according to the prediction using TargetScan, starBase, miRDB, and miRWalk (Figure 1A). Meanwhile, it was also predicted that LGG patients with high expression levels of miR‐942‐5p and miR‐455‐5p had poor survival (Figure 1B,C). GBM patients with high expression of miR‐942‐5p had poor survival, whereas those with high expression of miR‐455‐5p had better survival, as shown in Figure S1A,B. In addition, cBioPortal was exploited to analyze the correlation between miR‐942‐5p and miR‐455‐5p with prognosis. The results revealed that the neoplasm histologic grade, and new neoplasm event post‐initial therapy in miR‐942‐5p in Shallow Deletion and Diploid groups were promoted compared with those in Deep Deletion group (Figure 1D,E). Moreover, cBioPortal also showed that miR‐942‐5p and miR‐455‐5p in Shallow Deletion, Diploid, and Gain groups were linked to overall survival (Figure S1C,D). The levels of *KNG1*, miR‐942‐5p, and miR‐455‐5p in cancer lesions and adjacent normal samples from glioma patients were then evaluated (Figure 1F–I). As exhibited in Figure 1F,G, the low level of *KNG1* in glioma was both predicted through bioinformatics analysis (Figure 1G) and verified in clinical tissues (Figure 1F). The results of immunohistochemistry unveiled low expression of KNG1 in glioma samples (Figure S1E). Besides, miR‐942‐5p was highly expressed in glioma tissues (*p* < 0.001, Figure 1H), while no difference in miR‐455‐5p level was observed between cancer tissues and normal tissues (*p* > 0.05, Figure 1I). Moreover, our results uncovered a negative correlation between the expressions of *KNG1* and miR‐942‐5p in both normal tissues (*r* = −0.403, *p* = 0.003) and cancer tissues (*r* = −0.447, *p* < 0.001) (Figure 1J,K), whereas no correlation existed between miR‐455‐5p and *KNG1* (Figure 1L,M). Correspondingly, the transcription and translation levels of *KNG1* in glioma cells were remarkably decreased compared with those in NHA cells (*p* < 0.001, Figure 1N,O), while the level of miR‐942‐5p in glioma cells was signally enhanced (*p* < 0.05, Figure 1P). Given that *KNG1* had the lowest expression in LN‐229 cells and the highest expression in T98G cells, these two cell lines were singled out for later assays.

### 3.2 Silenced KNG1 enhanced the migration, invasion, and proliferation of LN‐229 and T98G cells, whereas KNG1 overexpression did the opposite

**FIGURE 2:**
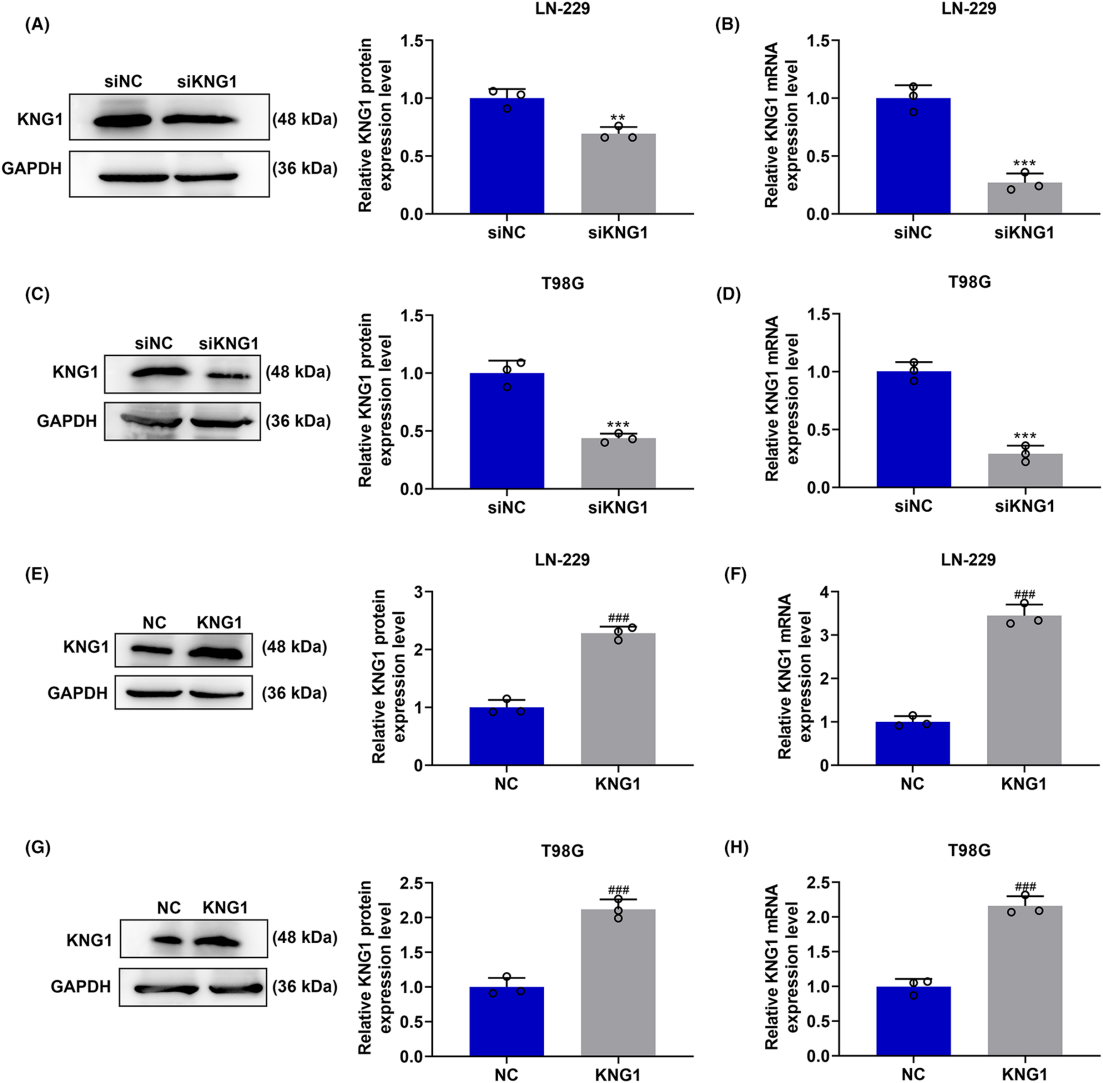
The transfection efficiency of siKNG1 and KNG1 overexpression plasmids in LN‐229 and T98G cells. (A–H) The expression level of *KNG1* in LN‐229 and T98G cells after transfection was detected by RT‐qPCR and Western blot. GAPDH was used as an internal control. (***p < 0.001 vs. siNC; ^###^ *p* < 0.001 vs. NC). All experiments were conducted three times. (NC: negative control)

**FIGURE 3:**
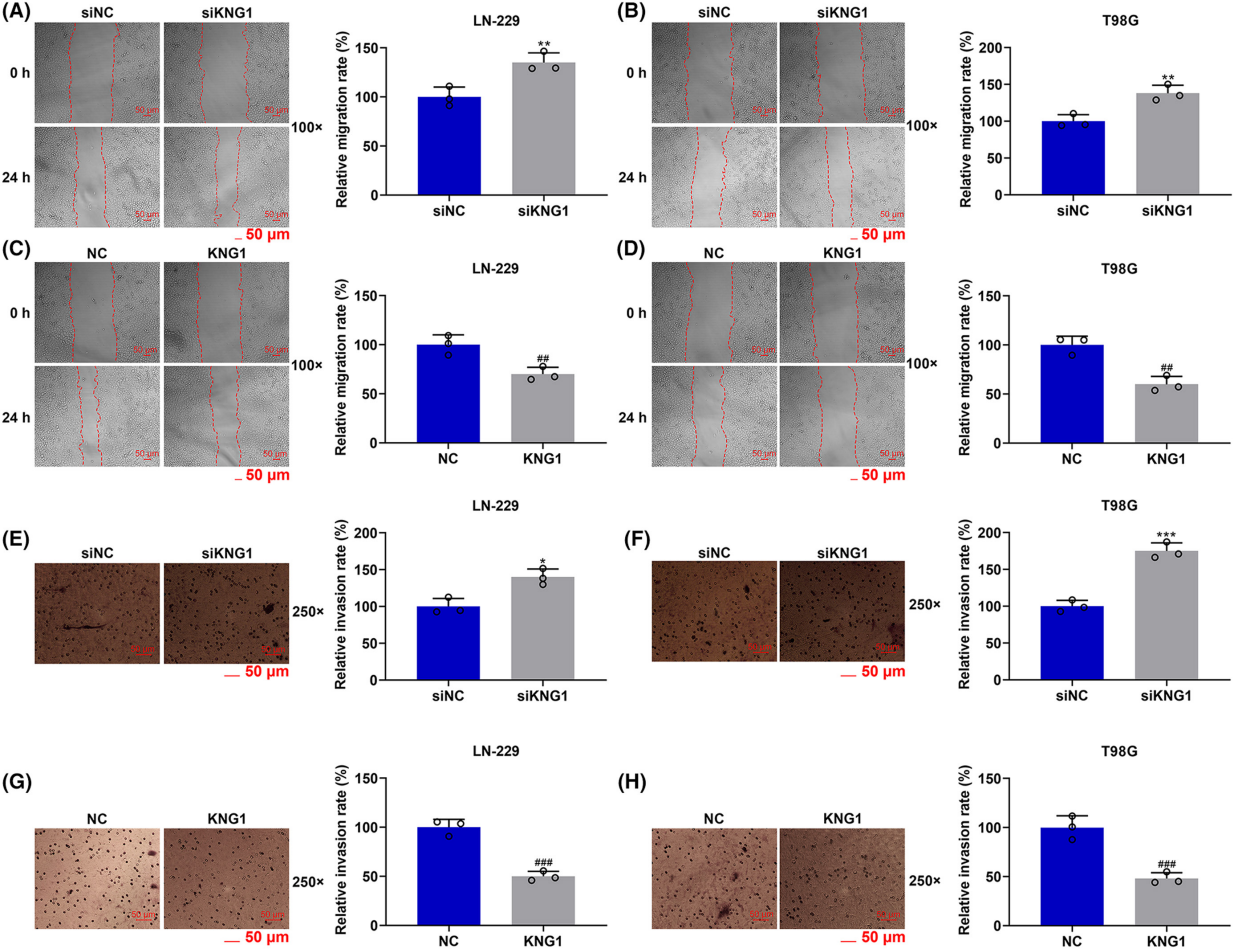
Silenced KNG1 enhanced the migration and invasion of LN‐229 and T98G cells, whereas KNG1 overexpression did the opposite. (A–D) The migration of LN‐229 and T98G cells after transfection of siKNG1 and KNG1 overexpression plasmids was determined by wound healing assays. (E–H) The invasion of LN‐229 and T98G cells after knockdown and overexpression of *KNG1* was detected by transwell assays (*p < 0.05, **p < 0.01, ***p < 0.001 vs. siNC; ^##^ *p* < 0.01, ^###^ *p* < 0.001 vs. NC). (NC: negative control)

**FIGURE 4:**
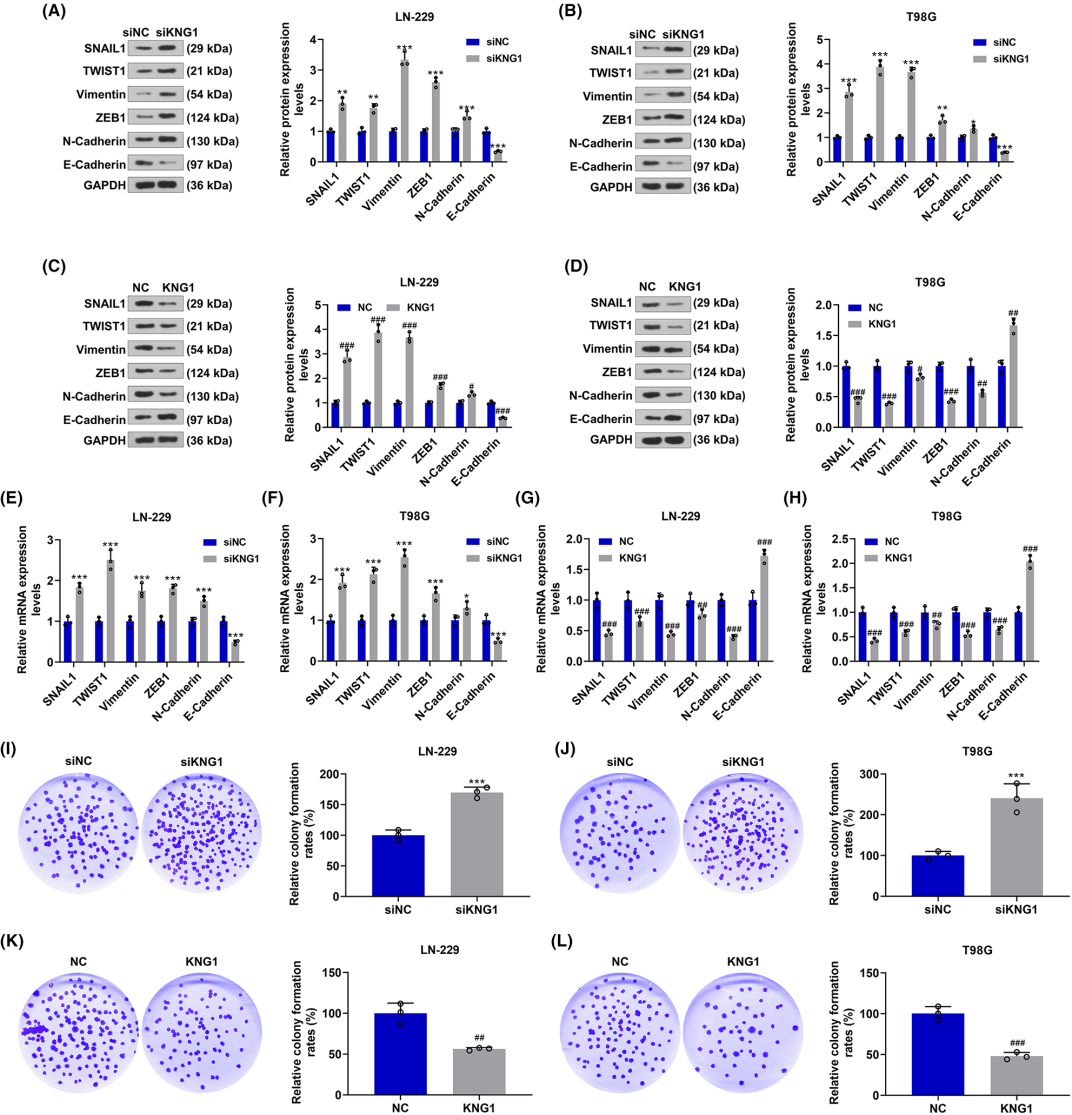
Silenced KNG1 facilitated the proliferation of LN‐229 and T98G cells by regulating related genes, whereas KNG1 overexpression did conversely. (A–H) The expressions of SNAIL1, TWIST1, Vimentin, ZEB1, N‐Cadherin, and E‐Cadherin in LN‐229 and T98G cells after knockdown and overexpression of *KNG1* were quantitated by RT‐qPCR or Western blot, with GAPDH serving as an internal control. (I–L) The proliferation of LN‐229 and T98G cells after knockdown and overexpression of *KNG1* was detected by colony formation assays (*p < 0.05, **p < 0.01, ***p < 0.001 vs. siNC; ^#^ *p* < 0.05, ^##^ *p* < 0.01, ^###^ *p* < 0.001 vs. NC) (NC: negative control)

*KNG1* was silenced or overexpressed in T98G and LN‐229 cells to determine its function in glioma. The transfection efficiency was detected by RT‐qPCR and Western blot analysis. The transcription and translation levels of *KNG1* were notably inhibited after transfection of siKNG1 into LN‐229 and T98G cells, compared with those in cells transfected with siNC (*p* < 0.01, Figure 2A–D), whereas the opposite tendencies (more than twofold levels) were observed after the transfection of KNG1 overexpression plasmids, compared with those in cells without treatment (*p* < 0.001, Figure 2E–H). Afterward, we detected the effects of *KNG1* on the migration and invasion abilities of LN‐229 and T98G cells (Figure 3A–H). The results indicated that the migration (Figure 3A,B) and invasion (Figure 3E,F) rates of LN‐229 and T98G cells were both significantly increased after transfection of siKNG1 (*p* < 0.05), but were both decreased after transfection of KNG1 overexpression plasmids (*p* < 0.01, Figure 3C,D,G,H). Subsequently, the levels of related genes were further determined to validate the above findings (4A‐H). As displayed in Figure 4A,B,E,F, in both LN‐229 and T98G cells transfected with siKNG1, the expressions of SNAIL1, TWIST1, Vimentin, ZEB1, and N‐Cadherin were upregulated, and that of E‐Cadherin was obviously down‐regulated (*p* < 0.05). By contrast, Figure 4C,D,G,H revealed that KNG1 overexpression generated the opposite effects on the expressions of the above genes in both cells. The changes in the proliferation of LN‐229 and T98G cells were also determined. As depicted in Figure 4I–L, transfection of siKNG1 caused evidently increased colony formation rate of the two cells (*p* < 0.001), while transfection of KNG1 overexpression plasmids brought about an obvious reduction in colony formation rate (*p* < 0.01). All these results signified that siKNG1 enhanced the malignant biological activities of LN‐229 and T98G cells, whereas KNG1 overexpression did the opposite.

### 3.3 MiR‐942‐5p targeted *KNG1* and regulated the expression of *KNG1*

**FIGURE 5:**
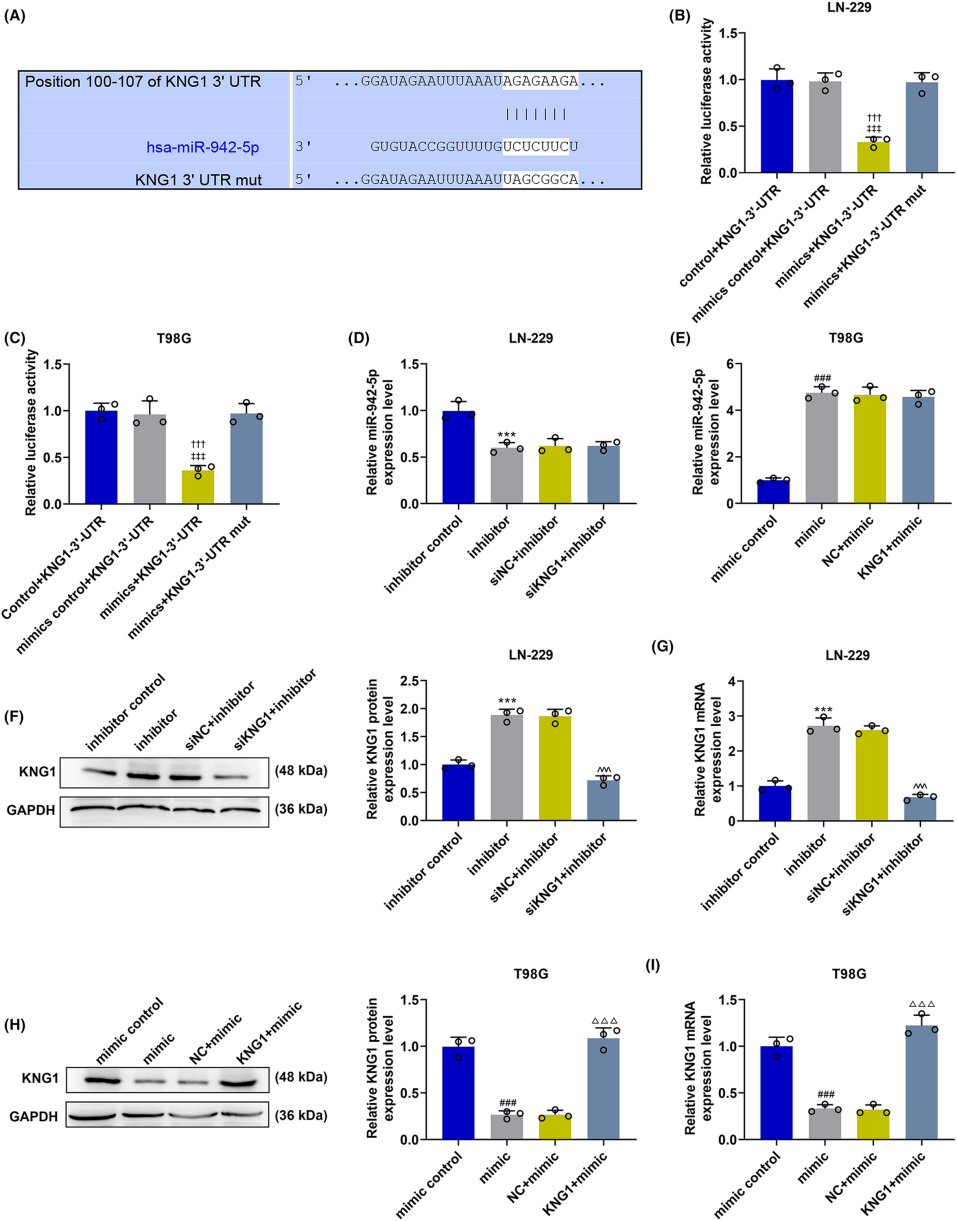
MiR‐942‐5p targeted *KNG1* and regulated the expression of *KNG1*. (A) KNG1‐3’‐UTR was predicted to contain binding sites of miR‐942‐5p by TargetScan. (B, C) Luciferase reporter assay validated that miR‐942‐5p targeted *KNG1* in LN‐229 and T98G cells (^‡‡‡^ *p* < 0.001 vs. mimics control+KNG1‐3’‐UTR, ^†††^ *P* < 0.001 vs. mimics + KNG1‐3’‐UTR mut). (D, E) The expression of miR‐942‐5p in LN‐229 and T98G cells after transfection was detected by RT‐qPCR, with U6 serving as an internal control. (F–I) The expression of *KNG1* in LN‐229 and T98G cells after transfection was detected by Western blot and RT‐qPCR, with GAPDH serving as an internal control. (***p < 0.001 vs. inhibitor control; ^^^^^ *p* < 0.001 vs. siNC + inhibitor; ^###^ *p* < 0.001 vs. mimic control; ^△△△^ *p* < 0.001 vs. NC + mimic) (NC: negative control)

After verification of the inhibitory effects of *KNG1* on proliferation, migration, and invasion of T98G and LN‐229 glioma cells, the miRNA‐targeting *KNG1* was detected. Targetscan predicted that *KNG1* might be targeted by miR‐942‐5p because KNG1‐3’‐UTR contained a target base sequence of miR‐942‐5p (Figure 5A). Then we conducted dual‐luciferase reporter assays to verify this prediction (Figure 5B,C). The findings demonstrated that the luciferase activity was prominently suppressed in the mimic + KNG1‐3’‐UTR group compared with that in the mimic control + KNG1‐3’‐UTR group, while no change of the luciferase activity was observed in the mimic + KNG1‐3’‐UTR mut group (*p* < 0.001). We also determined the changes in miR‐942‐5p expression after transfection (Figure 5D,E) and found that the level of miR‐942‐5p was markedly downregulated after transfection of miR‐942‐5p inhibitor yet appreciably upregulated by about twofold after transfection of miR‐942‐5p mimic (*p* < 0.001). However, no obvious change of miR‐942‐5p level was observed after co‐transfection of siKNG1 and miR‐942‐5p inhibitor or co‐transfection of KNG1 and miR‐942‐5p mimic, which indicated that *KNG1* cannot influence the expression of miR‐942‐5p. As depicted in Figure 5F,G, miR‐942‐5p inhibitor markedly upregulated the protein and mRNA levels of *KNG1* (*p* < 0.001). However, the promoting effect of miR‐942‐5p inhibitor was reversed by siKNG1 in comparison with siNC (*p* < 0.001). Besides, it can be noted in Figure 5H,I that miR‐942‐5p mimic dramatically reduced the protein and mRNA levels of *KNG1* (*p* < 0.001), which was reversed by KNG1 overexpression (*p* < 0.001). All these findings manifested that miR‐942‐5p targeted *KNG1* and regulated the expression of *KNG1*.

### 3.4 KNG1 overexpression and KNG1 knockdown counteracted the regulatory effects of miR‐942‐5p mimic and inhibitor on the migration, invasion, and proliferation of LN‐229 and T98G cells, respectively

**FIGURE 6:**
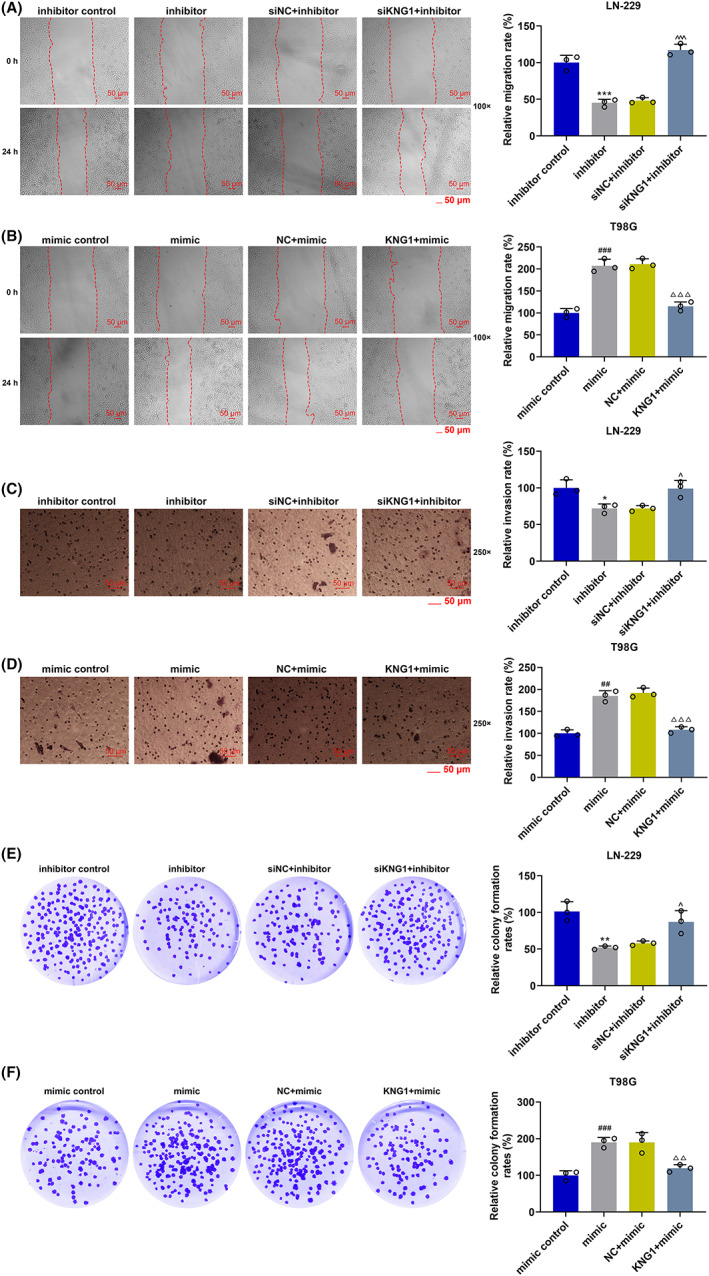
KNG1 reversed the promoting effect of miR‐942‐5p mimic on the migration, invasion, and proliferation of LN‐229 and T98G cells, and siKNG1 offset the inhibitory effect of miR‐942‐5p inhibitor on the migration and invasion of both cells. (A, B) The migration of LN‐229 and T98G cells after transfection was detected by wound healing assays. (C, D) The invasion of LN‐229 and T98G cells after transfection was detected by Transwell assays. (E, F) The proliferation of LN‐229 and T98G cells after transfection was detected by colony formation assays (*p < 0.05, **p < 0.01, ***p < 0.001 vs. inhibitor control; ^^^ *p* < 0.05, ^^^^ *p* < 0.01, ^^^^^ *p* < 0.001 vs. siNC + inhibitor; ^##^ *p* < 0.01, ^###^ *p* < 0.001 vs. mimic control; ^△△^ *p* < 0.01, ^△△△^ *p* < 0.001 vs. NC + mimic) (NC: negative control)

The impacts of *KNG1* combined with miR‐942‐5p on glioma cells were gauged by detecting changes in migration, invasion, and proliferation rates. As depicted in Figure 6A,C,E, the migration, invasion, and colony formation rates of LN‐229 cells were starkly lessened by an miR‐942‐5p inhibitor (*p* < 0.05), the trends of which were notably reversed by siKNG1 (*p* < 0.05). Meanwhile, as exhibited in Figure 6B,D,F, the migration, invasion, and colony formation rates of T98G cells were conspicuously elevated by miR‐942‐5p mimic (*p* < 0.01), the trends of which were explicitly reversed by KNG1 (*p* < 0.001). All these discoveries implied that miR‐942‐5p regulated the malignant biological activities of glioma cells by targeting *KNG1*.

### 3.5 *LINC01018* which was lowly expressed in glioma specifically targeted miR‐942‐5p

**FIGURE 7:**
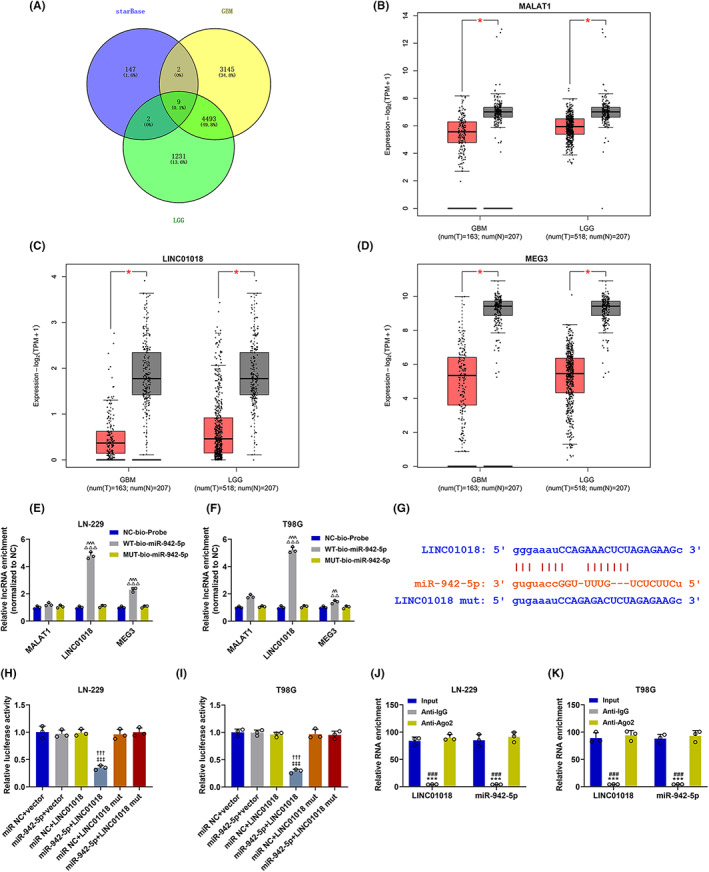
*LINC01018* was lowly expressed in glioma specifically targeted miR‐942‐5p. (A) The lncRNAs which might target miR‐942‐5p were predicted through bioinformatics analysis. (B–D) The expressions of *MALAT1* (B), *LINC01018* (C), and *MEG3* (D) in glioma were predicted through bioinformatics analysis. (E, F) RNA pull‐down assays were performed to detect whether miR‐942‐5p could bind with *LINC01018* and *MEG3*. (G) The putative binding site between *LINC01018* and miR‐942‐5p was predicted by starBase. (H–K) Dual‐luciferase reporter assay (H, I) and RIP assay (J, K) validated that *LINC01018* bound to miR‐942‐5p in LN‐229 and T98G cells (^‡‡‡^ *p* < 0.001 vs. miR NC + LINC01018, ^†††^ *p* < 0.001 vs. miR‐942‐5p + LINC01018 mut, ***p < 0.001 vs. Input; ^###^ *p* < 0.001 vs. Anti‐IgG) (NC: negative control, RIP: RNA immunoprecipitation)

The lncRNA which targeted miR‐942‐5p/*KNG1* axis and functioned in glioma was verified. Through analyzing the miRNA–lncRNA interactions supported by Ago CLIP‐seq Data,^30^ nine candidate lncRNAs were obtained. Among them, the expressions of *MALAT1*, *LINC01018*, and *MEG3* were lower in glioma (Figure 7B–D), and the three were discovered to be the lncRNAs that possibly targeted miR‐942‐5p (Figure 7A). RNA pull‐down assay results confirmed that miR‐942‐5p could bind with *LINC01018* and *MEG3* in both LN‐229 and T98G cells (Figure 7E, F). Considering that the binding of miR‐942‐5p to *LINC01018* was stronger than that to *MEG3*, *LINC01018* was singled out for later application. After that, the data from starBase v2.0 predicted that binding sites existed between miR‐942‐5p and *LINC01018* (Figure 7G). To verify this prediction, dual‐luciferase reporter assays were performed (Figure 7H,I). The results evidenced that luciferase activity was diminished in the miR‐942‐5p + LINC01018 group compared with that in the miR NC + LINC01018 group and the miR‐942‐5p + LINC01018 mut group (*p* < 0.001), while luciferase activity was not changed in miR‐942‐5p + LINC01018 mut group in contrast with that in the miR NC + LINC01018 mut group, implying that *LINC01018* could bind to miR‐942‐5p. Furthermore, we also used RIP to further verify whether *LINC01018* could target miR‐942‐5p (Figure 7J,K). The results demonstrated that *LINC01018* and miR‐942‐5p were precipitated in the Anti‐IgG group, relative to those in the Input group and the Anti‐Ago2 group (*p* < 0.001), proving that miR‐942‐5p could be directly targeted by *LINC01018*.

### 3.6 LINC01018 overexpression and knockdown reversed the regulatory effects of miR‐942‐5p mimic and inhibitor on the migration and invasion of LN‐229 and T98G cells, respectively

**FIGURE 8:**
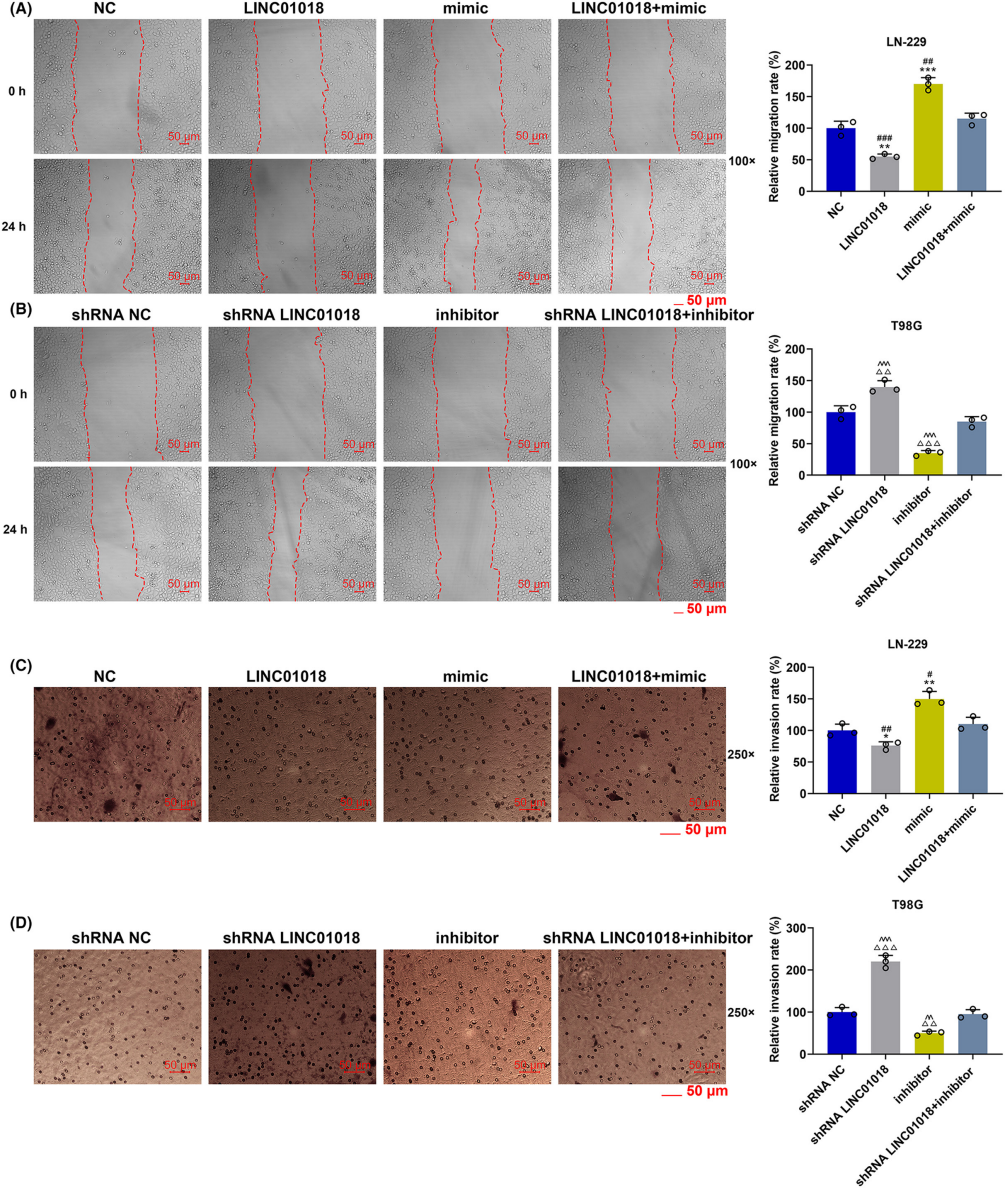
LINC01018 overexpression and knockdown reversed the regulatory effects of miR‐942‐5p mimic and inhibitor on the migration and invasion of LN‐229 and T98G cells, respectively. (A, B) The migration of LN‐229 and T98G cells after transfection was determined by wound healing assays. (C, D) The invasion of LN‐229 and T98G cells after transfection was detected by Transwell assays (*p < 0.05, **p < 0.01, ***p < 0.001 vs. NC; ^#^ *p* < 0.05, ^##^ *p* < 0.01, ^###^ *p* < 0.001 vs. LINC01018 + mimic; ^△△^ *p* < 0.01, ^△△△^ *p* < 0.001 vs. shRNA NC; ^^^^ *p* < 0.01, ^^^^^ *p* < 0.001 vs. shRNA LINC01018 + inhibitor). (NC: negative control)

**FIGURE 9:**
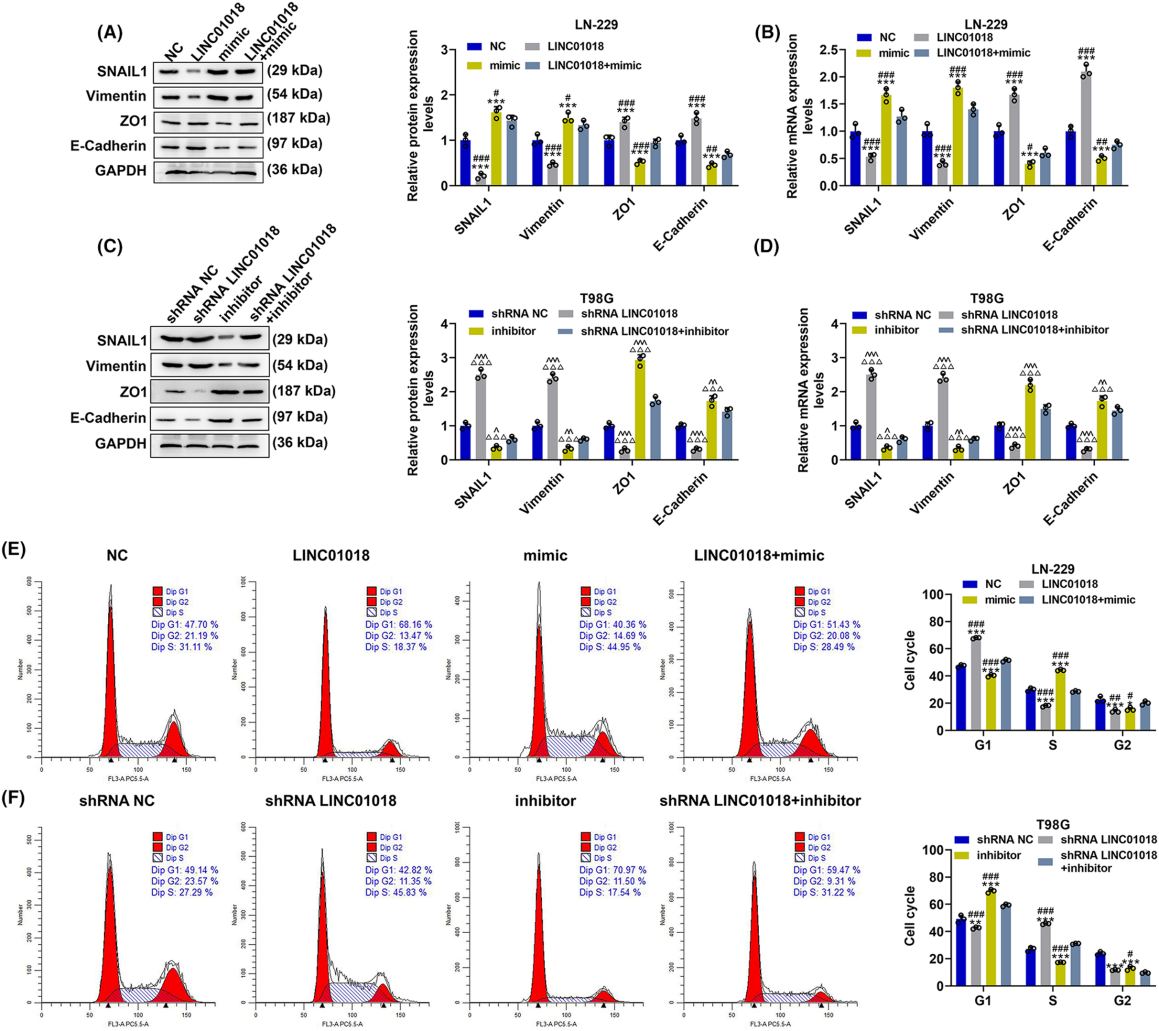
LINC01018 overexpression and knockdown diminished the effects of miR‐942‐5p mimic and inhibitor on cell cycle distribution and related gene expressions in LN‐229 and T98G cells, respectively. (A–D) The expressions of SNAIL1, Vimentin, ZO1, and E‐Cadherin in LN‐229 and T98G cells after transfection were quantified by Western blot and RT‐qPCR, with GAPDH serving as an internal control. (E, F) The cell cycle distribution of LN‐229 and T98G cells after transfection were detected by flow cytometry (*p < 0.05, **p < 0.01, ***p < 0.001 vs. NC; ^#^ *p* < 0.05, ^##^ *p* < 0.01, ^###^ *p* < 0.001 vs. LINC01018 + mimic; ^△^ *p* < 0.05, ^△△△^ *p* < 0.001 vs. shRNA NC; ^^^ *p* < 0.05, ^^^^ *p* < 0.01, ^^^^^ *p* < 0.001 vs. shRNA LINC01018 + inhibitor) (NC: negative control)

The effects and molecular mechanisms of *LINC01018*/miR‐942‐5p/*KNG1* axis on cell migration and invasion were detected. As Figure 8A,C mirrored, the migration and invasion rates of LN‐229 cells were distinctly diminished by LINC01018 overexpression but were notably augmented by miR‐942‐5p mimic (*p* < 0.05). Figure 8B,D revealed that both rates of T98G cells were markedly inhibited by miR‐942‐5p inhibitor yet prominently promoted by shRNA of LINC01018 (*p* < 0.01). Additionally, the protein and gene expressions of invasion‐related factors including SNAIL1, Vimentin, and ZO1 were downregulated by LINC01018 overexpression and miR‐942‐5p inhibitor but were upregulated by shRNA of LINC01018 and miR‐942‐5p mimic (*p* < 0.05); however, the protein and gene levels of E‐cadherin showed the opposite changing tendency (Figure 9A–D). The above results affirmed that LINC01018 modulated the migration and invasion of glioma cells by targeting miR‐942‐5p.

### 3.7 LINC01018 overexpression and knockdown counteracted the regulatory effects of miR‐942‐5p mimic and inhibitor on the cell cycle distribution and proliferation of LN‐229 and T98G cells, respectively

**FIGURE 10:**
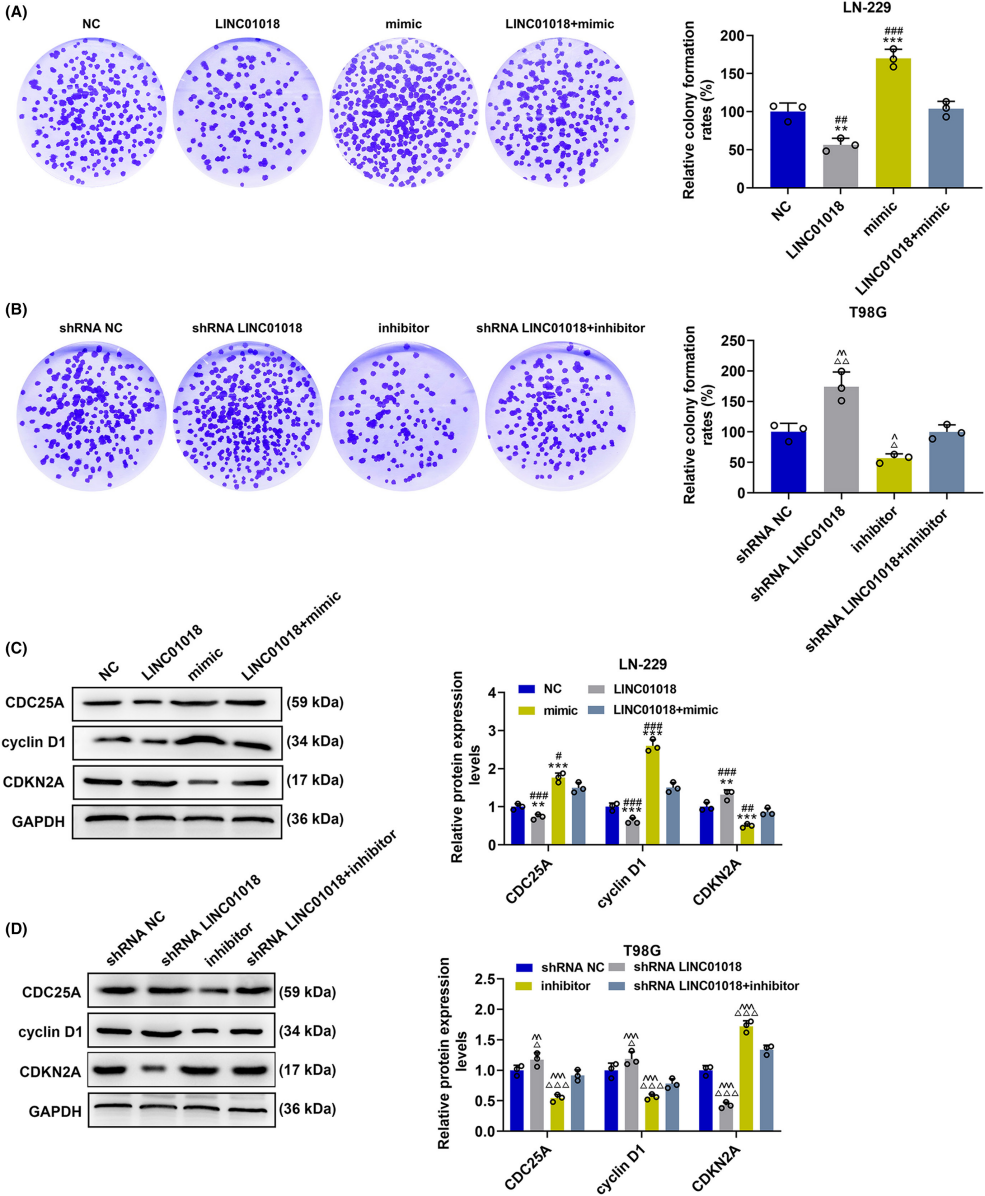
LINC01018 overexpression and knockdown reversed the effects of miR‐942‐5p mimic and inhibitor on the proliferation of LN‐229 and T98G cells, respectively. (A, B) The proliferation of LN‐229 and T98G cells after transfection was tested by colony formation assays. (C, D) The expressions of CDC25A, cyclin D1, and CDKN2A in LN‐229 and T98G cells after transfection were measured by Western blot, with GAPDH serving as an internal control (**p < 0.01, ***p < 0.001 vs. NC; ^#^ *p* < 0.05, ^##^ *p* < 0.01, ^###^ *p* < 0.001 vs. LINC01018 + mimic; ^△^ *p* < 0.05, ^△△^ *p* < 0.01, ^△△△^ *p* < 0.001 vs. shRNA NC; ^^^ *p* < 0.05, ^^^^ *p* < 0.01, ^^^^^ *p* < 0.001 vs. shRNA LINC01018 + inhibitor) (NC: negative control)

The effects and molecular mechanisms of *LINC01018*/miR‐942‐5p/*KNG1* axis on cell cycle and proliferation were tested. As detailed in Figure 9E, LINC01018 overexpression overtly augmented the proportion of LN229 cells in the G1 phase (*p* < 0.001) and obviously reduced that in the S phase (*p* < 0.05) and the G2 phase (*p* < 0.001). By contrast, miR‐942‐5p mimic significantly decreased the proportion of LN229 cells in the G1 phase (*p* < 0.01) and in the G2 phase (*p* < 0.05) and markedly increased the proportions of those in the S phase (*p* < 0.05). These results reflected that miR‐942‐5p mimic positively changed the cell cycle distribution of LN‐229 cells, whereas LINC01018 overexpression generated the opposite effect and could further reverse the positive effect of miR‐942‐5p mimic on cell cycle distribution. As illustrated in Figure 9F, shRNA of LINC01018 and miR‐942‐5p inhibitor possessed the opposite effects to LINC01018 overexpression and miR‐942‐5p mimic, respectively, and shRNA of LINC01018 neutralized the effect of miR‐942‐5p inhibitor on cell cycle distribution. As for cell proliferation (Figure 10A,B), the relative colony formation of LN‐229 cells was markedly hindered by LINC01018 overexpression and facilitated by miR‐942‐5p mimic (*p* < 0.01), while the relative colony formation of T98G cells was strongly boosted by shRNA of LINC01018 and blocked by an miR‐942‐5p inhibitor (*p* < 0.05). Also, the effects of miR‐942‐5p mimic and inhibitor on cell proliferation were offset by LINC01018 overexpression and knockdown, respectively. We then detected the expressions of proliferation‐related proteins (Figure 10C,D). LINC01018 overexpression notably reduced the expressions of CDC25A and cyclin D1 (*p* < 0.05) and evidently elevated that of CDKN2A (*p* < 0.01), while miR‐942‐5p mimic produced the inverse effects which could be reversed by LINC01018 overexpression. shRNA of LINC01018 and miR‐942‐5p inhibitor had the opposite effects to LINC01018 overexpression and miR‐942‐5p mimic on these proteins, respectively, and shRNA of LINC01018 could reverse the effect of miR‐942‐5p inhibitor on cell proliferation. All these discoveries reflected that *LINC01018* regulated the proliferation of glioma cells by targeting miR‐942‐5p.

### 3.8 *LINC01018* targeted miR‐942‐5p to regulate the tumor growth, *KNG1* expression, and MVD

**FIGURE 11:**
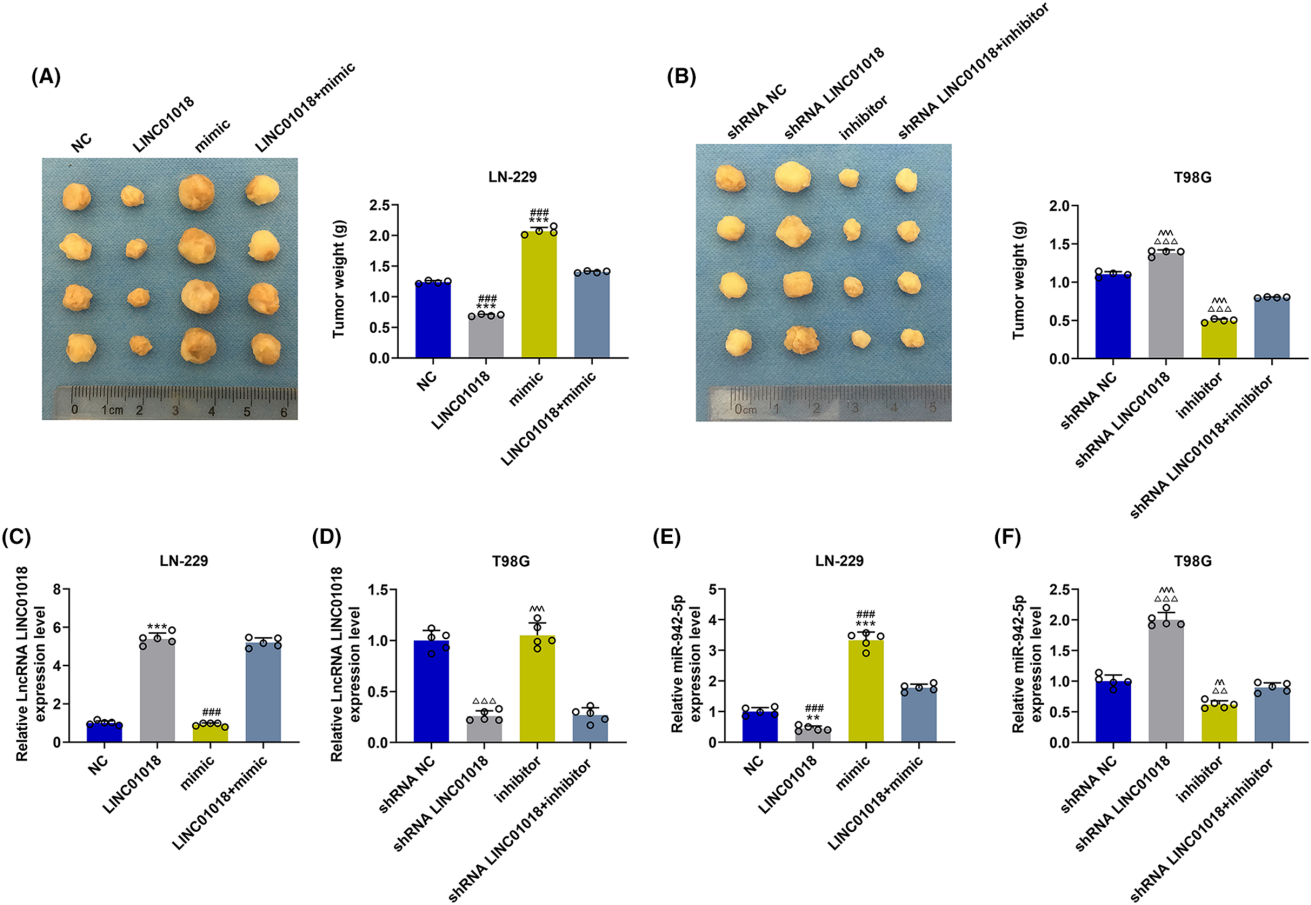
*LINC01018* targeted miR‐942‐5p to regulate tumor growth in vivo. (A, B) The picture of a solid tumor was exhibited and the tumor weight was calculated. (C, D) The expression of *LINC01018* in tumor tissues was measured by RT‐qPCR, with GAPDH serving as an internal control. (E, F) The expression of miR‐942‐5p in tumor tissues was detected by RT‐qPCR, with U6 serving as an internal control (**p < 0.01, ***p < 0.001 vs. NC; ^###^ *p* < 0.001 vs. LINC01018 + mimic; ^△△^ *p* < 0.01, ^△△△^ *p* < 0.001 vs. shRNA NC; ^^^^ *p* < 0.01, ^^^^^ *p* < 0.001 vs. shRNA LINC01018 + inhibitor) (NC: negative control)

**FIGURE 12:**
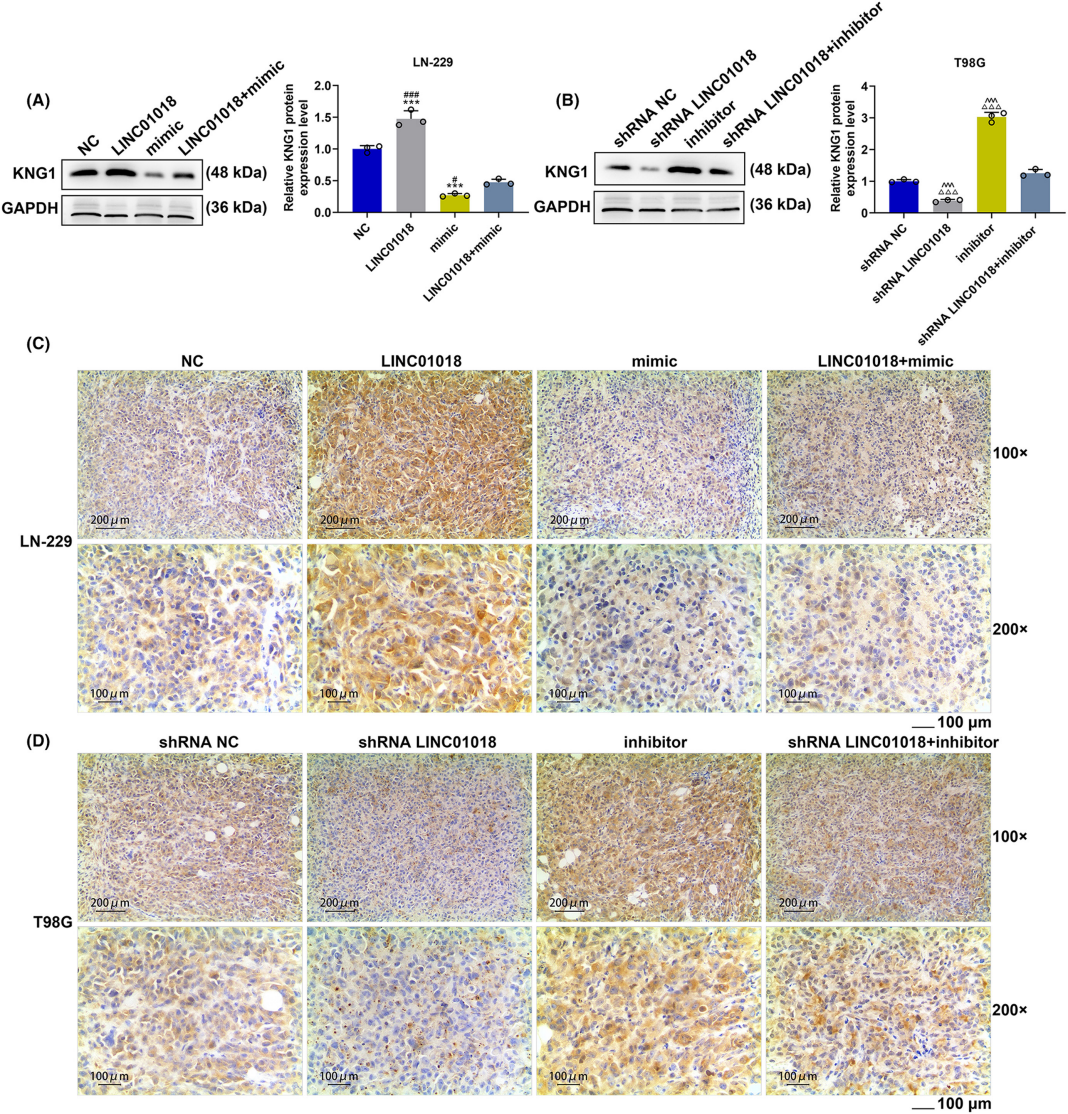
*LINC01018* targeted miR‐942‐5p to regulate the expression of *KNG1* in vivo. (A, B) The expression of *KNG1* in tumor tissues was quantitated by Western blot and RT‐qPCR, with GAPDH serving as an internal control. (C, D) The expression of *KNG1* in tumor tissues was determined by immunohistochemical analysis. (***p < 0.001 vs. NC; ^###^ *p* < 0.001 vs. LINC01018 + mimic; ^△△△^ *p* < 0.001 vs. shRNA NC; ^^^^^ *p* < 0.001 vs. shRNA LINC01018 + inhibitor). (NC: negative control)

**FIGURE 13:**
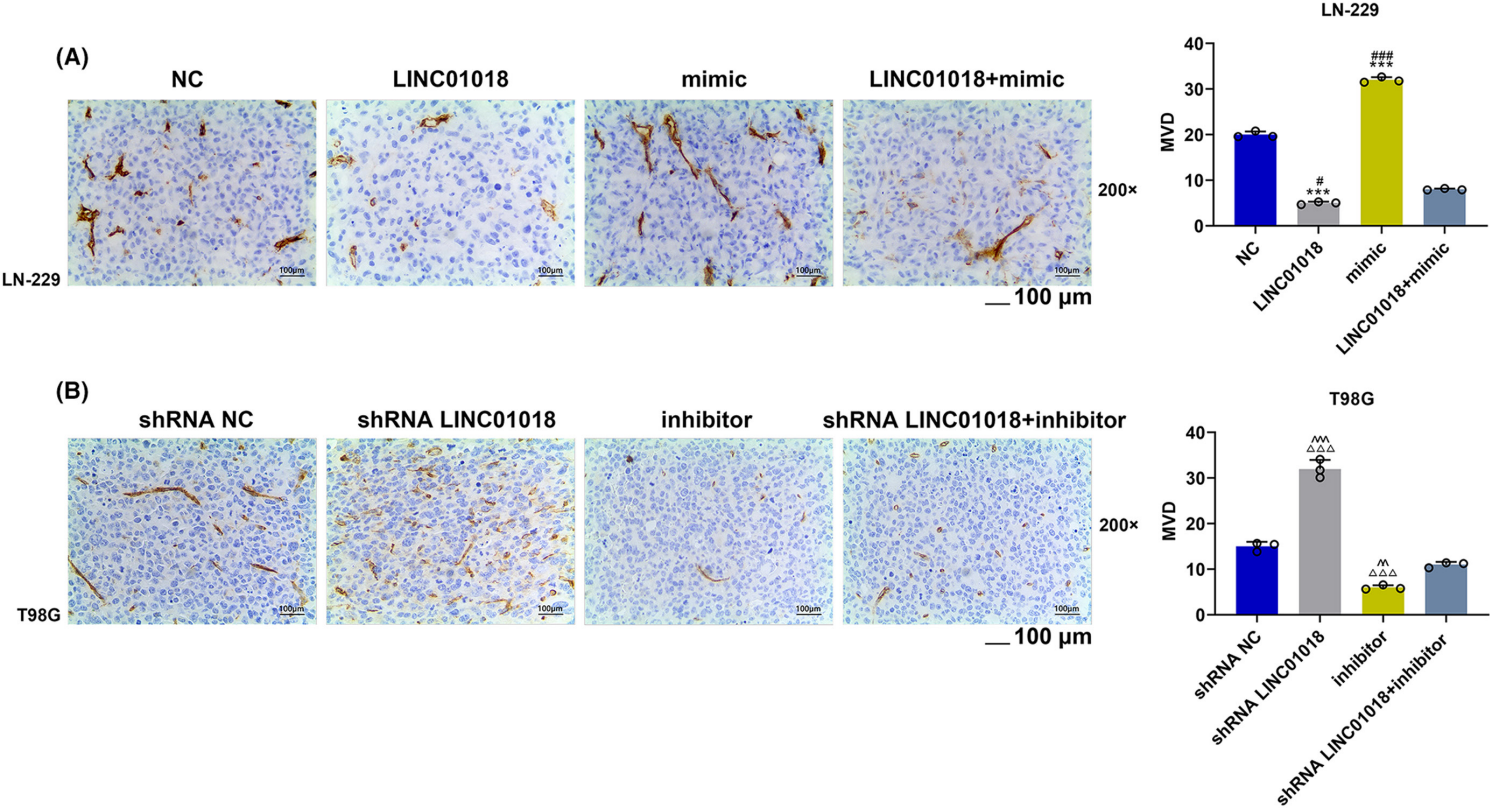
*LINC01018* targeted miR‐942‐5p to regulate the microvessel density (MVD). (A, B) The effect of *LINC01018/*miR‐942‐5p axis on MVD was analyzed by immunohistochemistry (***p < 0.001 vs. NC; ^#^ *p* < 0.05, ^###^ *P* < 0.001 vs. LINC01018 + mimic; ^△△△^ *p* < 0.001 vs. shRNA NC; ^^^^ *p* < 0.01, ^^^^^ *p* < 0.001 vs. shRNA LINC01018 + inhibitor) (NC: negative control)

Animal experiments were performed to verify the influence of *LINC01018‐*targeting miR‐942‐5p on glioma in vivo. In line with the tumor picture and the tumor weight statistical chart (Figure 11A,B), the tumor weight of mice injected with LN‐229 cells was reduced by LINC01018 overexpression but was elevated by miR‐942‐5p mimic, while the tumor weight of mice injected with T98G cells was decreased by miR‐942‐5p inhibitor but was increased by LINC01018 knockdown (*p* < 0.001). Also, the effects of miR‐942‐5p mimic were further reversed by LINC01018 overexpression in mice injected with LN‐229 cells, and those of miR‐942‐5p inhibitor were offset by LINC01018 knockdown in mice injected with T98G cells (*p* < 0.001). According to Figure 11C,D, the expression of LINC01018 was significantly upregulated by LINC01018 overexpression in LN‐229 cells but was downregulated by shRNA LINC01018 in T98G cells (*p* < 0.001). In LN‐229 cells, the expression of miR‐942‐5p (Figure 11E,F) was starkly dwindled by LINC01018 overexpression but was obviously elevated by miR‐942‐5p mimic. In T98G cells, the expression of miR‐942‐5p was remarkably inhibited by miR‐942‐5p inhibitor but was obviously promoted by LINC01018 knockdown (*p* < 0.01). Besides, the effects of miR‐942‐5p mimic were conspicuously reversed by LINC01018 overexpression in LN‐220 cells, while the effects of miR‐942‐5p inhibitor were offset by LINC01018 knockdown in T98G cells. We also detected the protein and gene expressions of *KNG1* (Figure 12A,B), with the results implicating that *KNG1* expression was explicitly upregulated by LINC01018 overexpression but was evidently downregulated by miR‐942‐5p mimic in LN‐229 cells, while *KNG1* expression was promoted by miR‐942‐5p inhibitor but was suppressed by LINC01018 knockdown in T98G cells (*p* < 0.01). Furthermore, the effect of miR‐942‐5p mimic on *KNG1* expression was significantly reversed by LINC01018 overexpression in LN‐229 cells, while that of miR‐942‐5p inhibitor on *KNG1* expression was reversed by LINC01018 knockdown in T98G cells. These results were also further verified by the data from immunohistochemistry (Figure 12C,D). Since KNG1 could suppress angiogenesis, immunohistochemistry was used to detect MVD. As per Figure 13A,B, we discovered that LINC01018 overexpression inhibited MVD, while LINC01018 knockdown did oppositely. Moreover, the effects of LINC01018 overexpression and knockdown on MVD were markedly neutralized by miR‐942‐5p mimic and inhibitor, respectively (*p* < 0.05).

## 4 DISCUSSION

*KNG1* has been identified as a biomarker for colorectal cancer, ovarian carcinoma, and many other cancers, owing to its ability in modulating the progression of a wide range of diseases and cancers.^8^, ^11^, ^12^ Recently, low expression of *KNG1* has been found in the serum of glioma patients.^13^ Consistently, we found that *KNG1* was lowly expressed in glioma tissues and cells. Our previous research also proved that *KNG1* overexpression could inhibit proliferation and induce apoptosis in glioma cells.^13^ In this study, the effects of *KNG1* on the biological functions of glioma cells were enriched, further suggesting the inhibitory effect of *KNG1* on the development of glioma.

Furthermore, there are no reports concerning the insights into the action mechanism of *KNG1* in glioma. As a common upstream regulatory gene of mRNA, miRNA has attracted our attention. Moreover, a quite few miRNA species have been suggested to be biomarkers for glioma.^32^, ^33^, ^34^ For instance, miR‐339/342 can be targeted by *FOXD1‐AS1* to regulate glioma biological processes.^34^ Therefore, further studies of miRNAs that target *KNG1* may provide new markers for the diagnosis and treatment of glioma. Here, we discovered that miR‐942‐5p and miR‐455‐5p might be the regulatory miRNAs of *KNG1*, and further revealed the correlation between the high survival of patients, and the low expression levels of miR‐942‐5p and miR‐455‐5p. Considering that miR‐455‐5p had no correlation with *KNG1*, miR‐942‐5p was chosen for later experiments. Previous research suggested that miR‐942‐5p is abnormally expressed in various types of diseases, including multiple sclerosis, breast cancer, and Kaposi's Sarcoma.^35^, ^36^, ^37^, ^38^ However, the effect of miR‐942‐5p on glioma needed more exploration. We uncovered that aberrant expression of miR‐942‐5p was related to glioma. One of the difficulties in treating glioma is the ability of glioma cells to rapidly proliferate and infiltrate normal tissues.^39^ In this part, we corroborated that miR‐942‐5p mimic could promote glioma cells to migrate, invade, and proliferate and that miR‐942‐5p inhibitor had an inhibitory effect on these biological functions of glioma cells. Besides, the effects of miR‐942‐5p mimic and inhibitor could be reversed by *KNG1* overexpression and knockdown, respectively, which unearthed that miR‐942‐5p regulated the malignant biological activities of glioma cells by targeting *KNG1*. Moreover, miR‐942‐5p has been found to participate in the ceRNA mechanism in various cancers. For instance, miR‐942‐5p is adsorbed by *lncRNA HCG11* and then targets growth factor‐independent transcription repressor 1 to block the progression of cervical cancer^40^; and *LIFR‐AS1* sponges miR‐942‐5p to increase the level of *ZNF471*, thereby inhibiting the malignant phenotype of nonsmall‐cell lung cancer.^41^ Therefore, fathoming out the upstream lncRNA of miR‐942‐5p is conducive to further understanding the mechanism of miR‐942‐5p/*KNG1* axis in glioma.

LncRNA, a kind of noncoding RNA, shares many features with mRNA and could competitively bind to miRNAs to regulate their downstream signal transduction.^25^, ^42^ The mechanisms were subsequently further illustrated in this study, as we additionally found that miR‐942‐5p could bind to *LINC01018* which is a newly identified lncRNA and plays an important regulatory role in liver cancer and liver diseases.^43^, ^44^ In line with previous research, *LINC01018* acts as a novel suppressor for liver cancer by dint of its inhibiting effect on proliferation and promoting effect on apoptosis of liver cancer cells via miR‐182‐5p^44^; also, it serves as a sponge of miRNA in cancers and modulates the expressions of genes involved in fatty acid oxidation in livers via interacting with HuR.^43^ Nonetheless, the effect of *LINC01018* on glioma has not been reported so far. Here, we discovered that *LINC01018* regulated the migration, invasion, proliferation, and cell cycle distribution of glioma cells by targeting miR‐942‐5p to alter epithelial‐mesenchymal transition (EMT) or proliferation‐related proteins.

In order to confirm the results in the present study in vivo, we further established the nude mice subcutaneous xenotransplanted tumor model, which is usually used to verify the effect of a certain gene on tumor growth.^45^ The results confirmed that *LINC01018* overexpression not only inhibited the growth of glioma cells but also downregulated miR‐942‐5p expression and upregulated *KNG1* expression, while *LINC01018* knockdown and miR‐942‐5p mimic did the opposite. In addition, the effects of miR‐942‐5p mimic and inhibitor on tumor growth were found to be neutralized by LINC01018 overexpression and knockdown, respectively, which further substantiated the results of in vitro assays. However, it is a pity that we did not construct an in vivo metastasis model to confirm our conclusions. In the future, the influence of *LINC01018*/miR‐942‐5p/*KNG1* axis on the local or distant invasion and metastasis of tumors in vivo may be further explored. Many modalities and methods have recently been developed for the in vivo evaluation of gliomas, including “ultramicroscopy” and magnetic resonance imaging.^46^, ^47^, ^48^ However, this study is currently limited to in vivo methods of RT‐PCR and immunohistochemistry and may be further explored in its future studies in combination with other non‐invasive research methods.

In a word, the results in this study authenticated that *LINC01018* overexpression could inhibit the proliferation, migration, and invasion of glioma cells and the growth of glioma in nude mice by targeting the miR‐942‐5p/*KNG1* axis. Our research has enriched the mechanism of glioma and laid the foundation for subsequent research.

Despite our extensive studies, some limitations still remain. Only one cell line is transplanted in the current in vivo study, and the cell‐to‐cell heterogeneity is evident based on previous single‐cell sequencing studies, which needs to be validated by increasing the volume of cell experiments in the future. TMZ is currently the first‐line anti‐glioma drug, and testing LINC01018/miR‐942‐5p/KNG1 in future studies using TMZ‐resistant/sensitive cells may also provide new evidence for the clinical application of this pathway. In addition, future studies may consider the noninvasive assessment of tumor metabolism.

## FUNDING INFORMATION

This work was supported by the National Natural Science Foundation of China (81702462) and the Zhejiang Natural Science Foundation Project (LY21H160024)

## CONFLICT OF INTEREST

The authors declare no conflict of interest.

## Supporting information


FigureS1

AppendixS1

## Data Availability

The analyzed data sets generated during the study are available from the corresponding author on reasonable request.
