# Supplementary figures and images for: LncRNA LINC01018/miR‐942‐5p/KNG1 axis regulates the malignant development of glioma in vitro and in vivo
Source: CNS Neurosci Ther. 2022 Dec 22;29(2):691–711. doi: 10.1111/cns.14053 (PMC9873518; doi:10.1111/cns.14053)

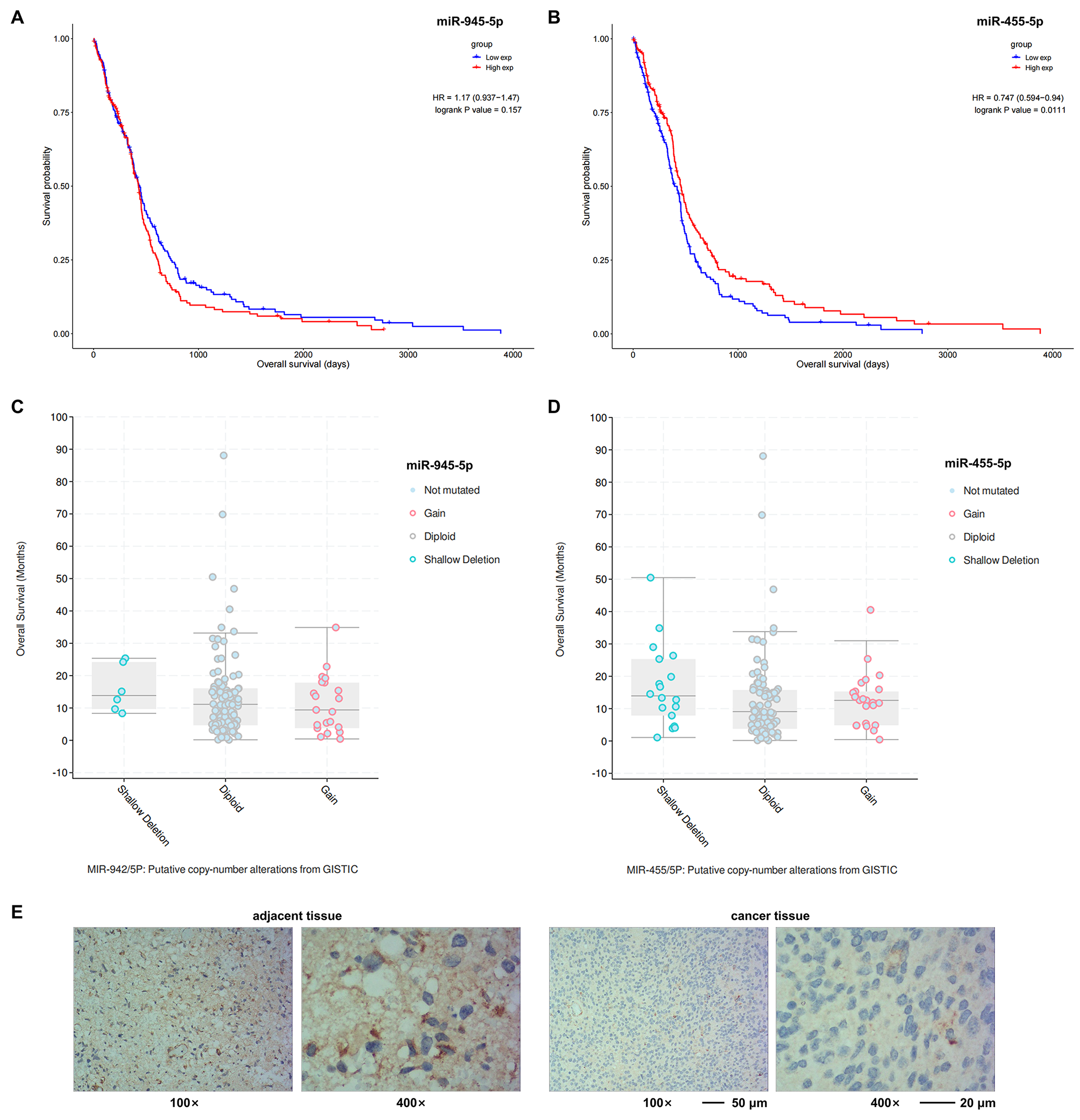

Supplement: Supplementary file 1 — FigureS1 [file CNS-29-691-s001.tif]
