# Supplementary material for: LncRNA LINC01018/miR‐942‐5p/KNG1 axis regulates the malignant development of glioma in vitro and in vivo
Source: CNS Neurosci Ther. 2022 Dec 22;29(2):691–711. doi: 10.1111/cns.14053 (PMC9873518; doi:10.1111/cns.14053)

Full unedited gel/blot for Figure 1

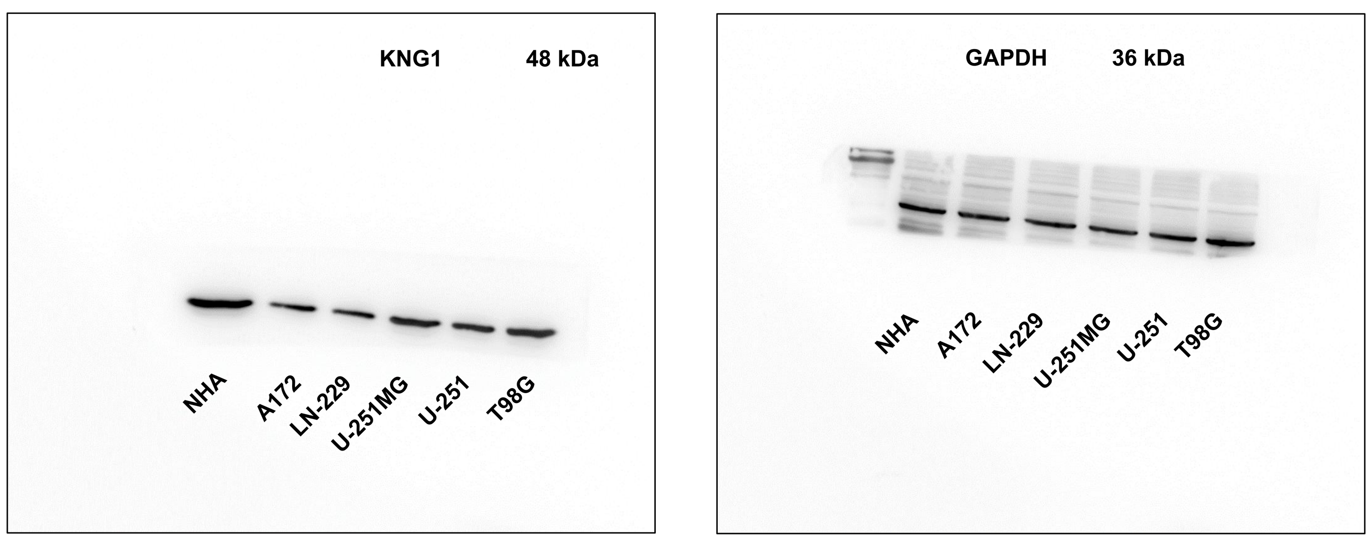

Full unedited gel/blot for Figure 2

LN-229 T98G

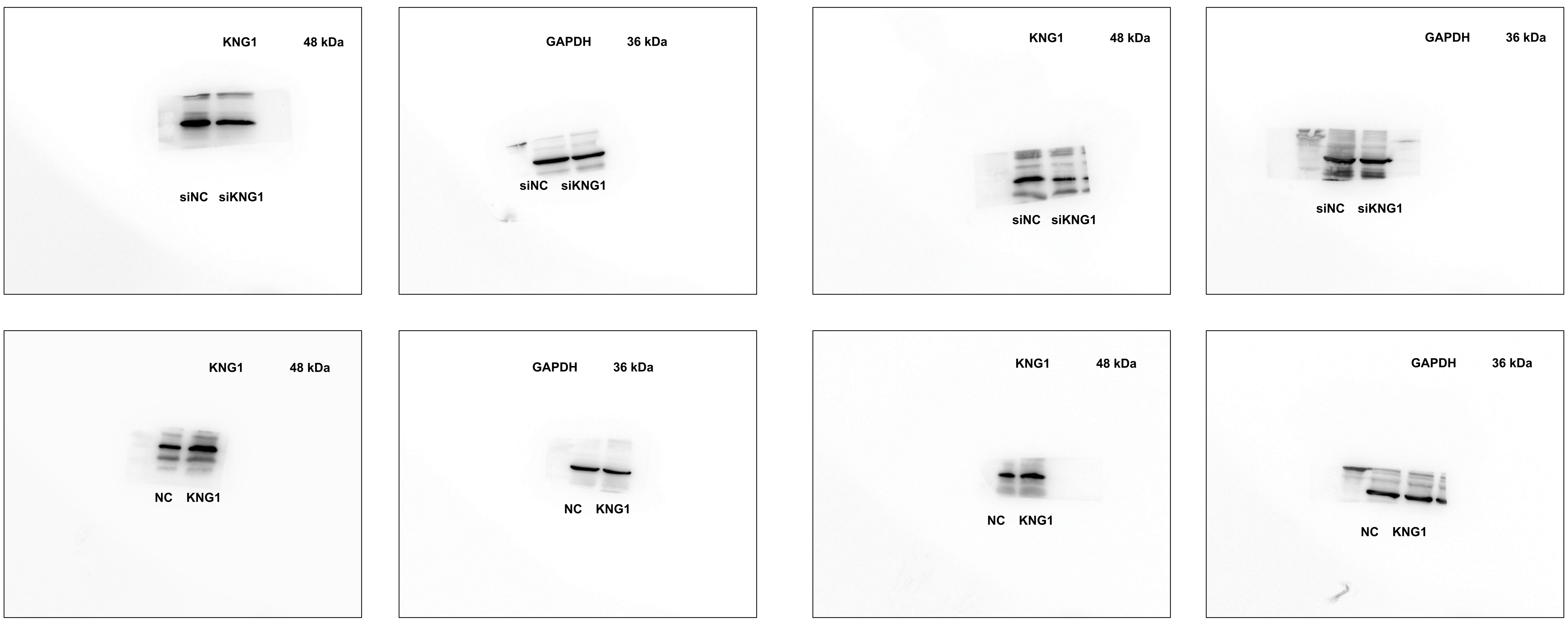

Full unedited gel/blot for Figure 4

LN-229

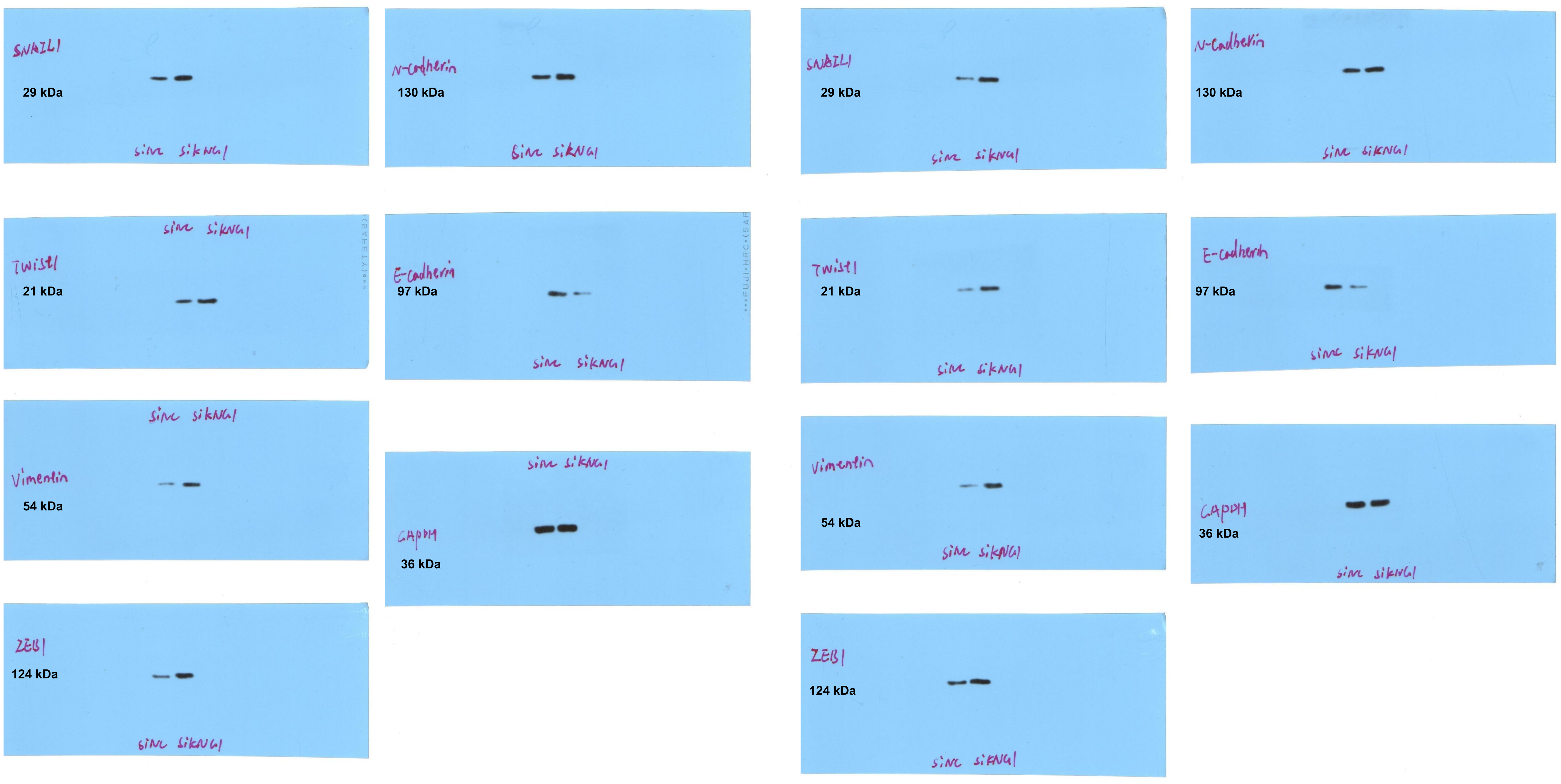

T98G

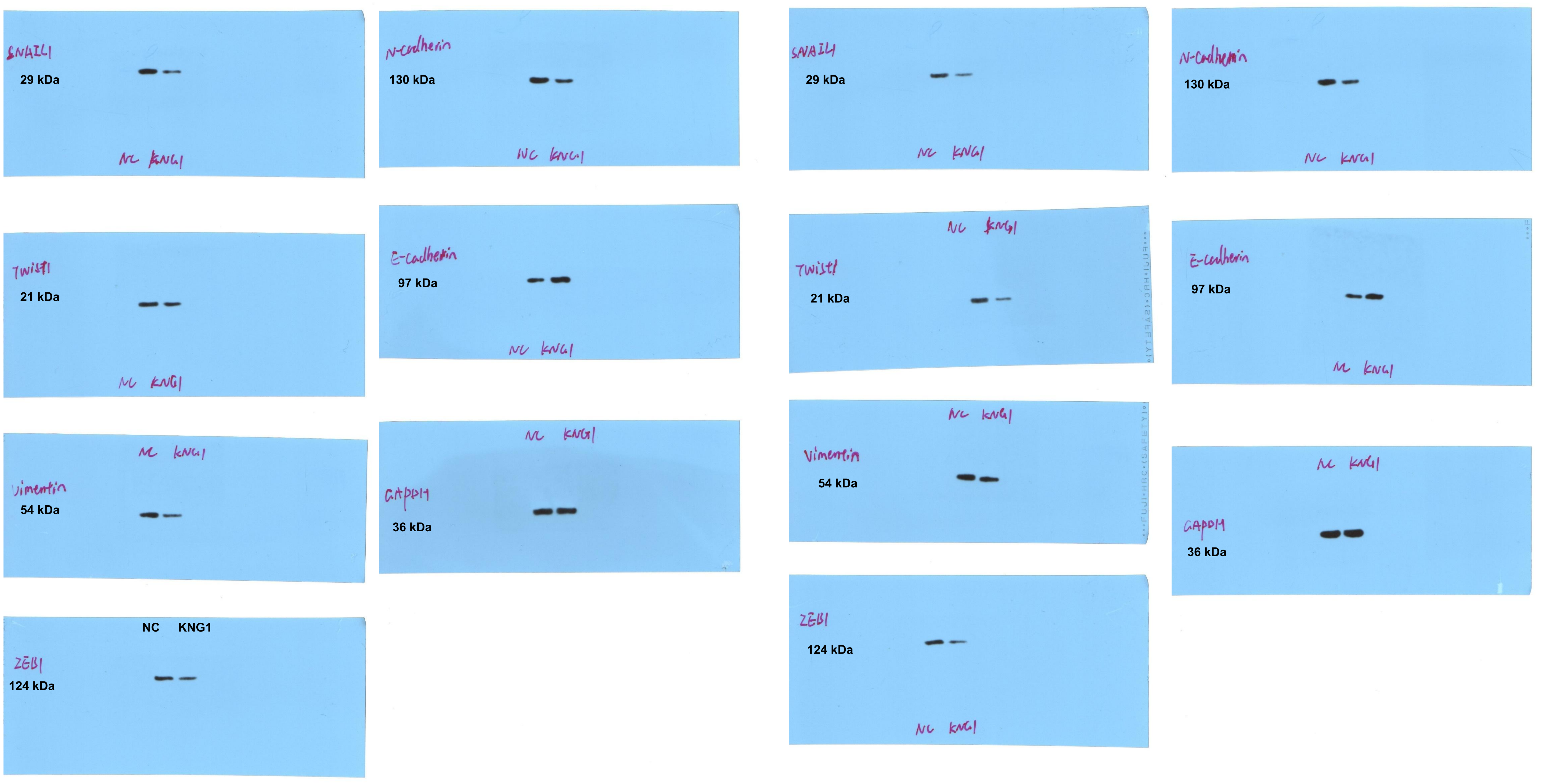

Full unedited gel/blot for Figure 5

LN-229 T98G

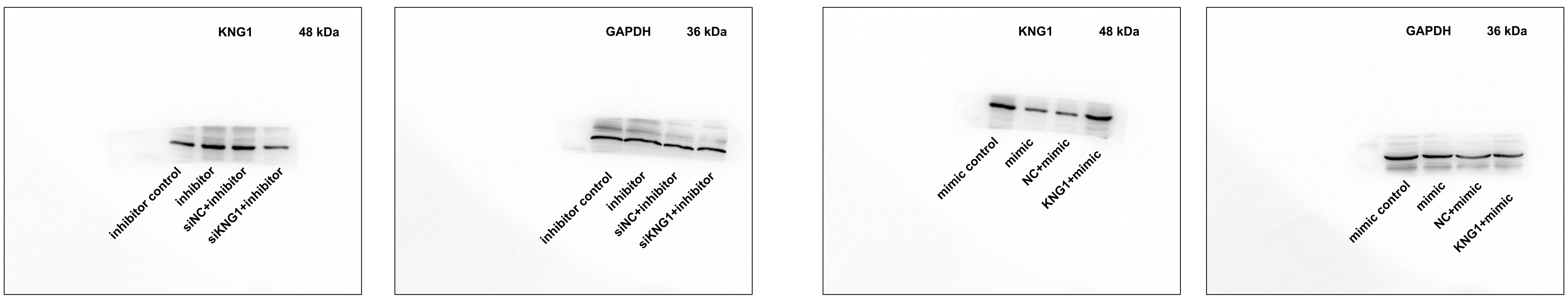

Full unedited gel/blot for Figure 9

LN-229 T98G

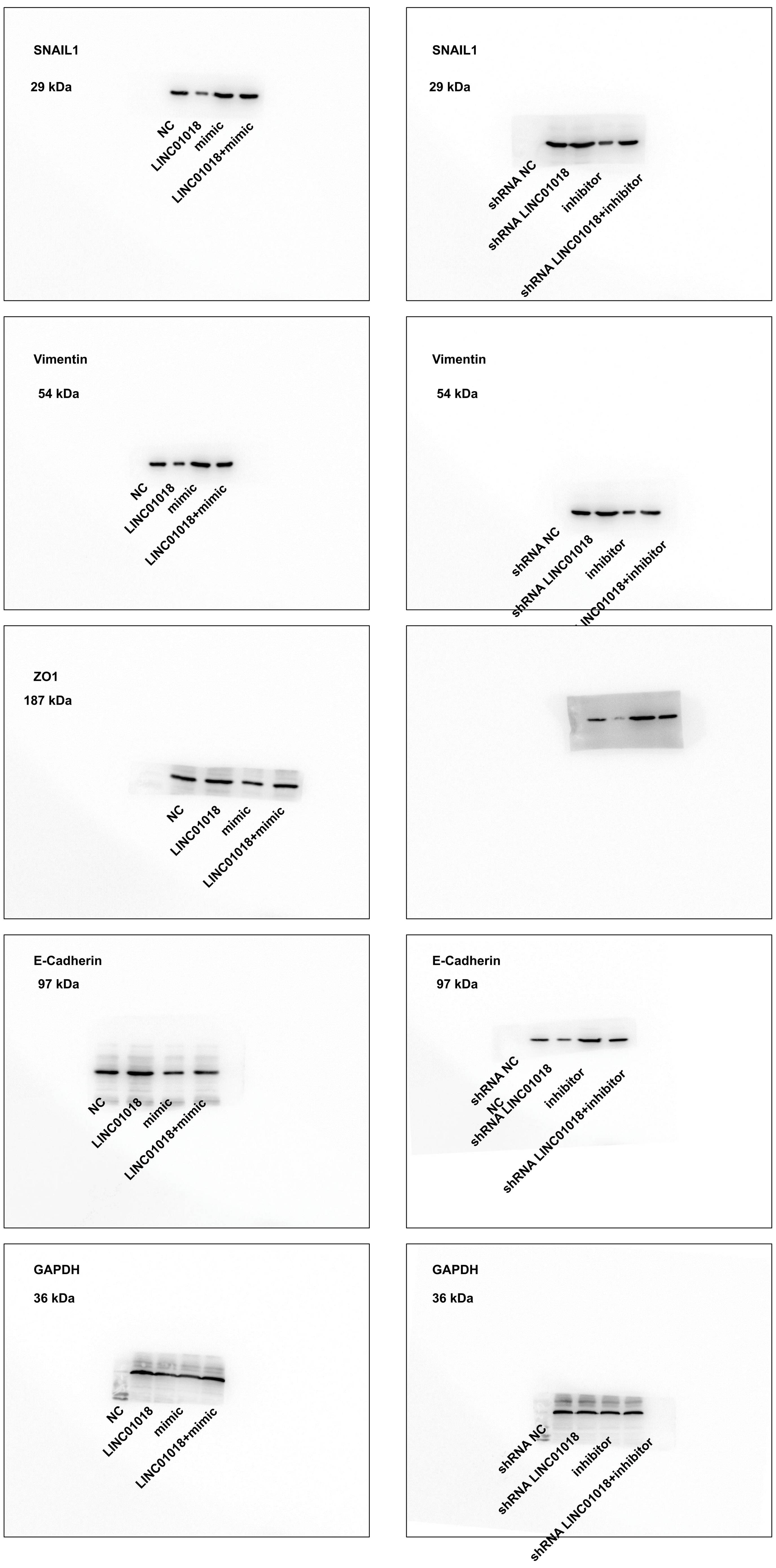

Full unedited gel/blot for Figure 10

LN-229 T98G

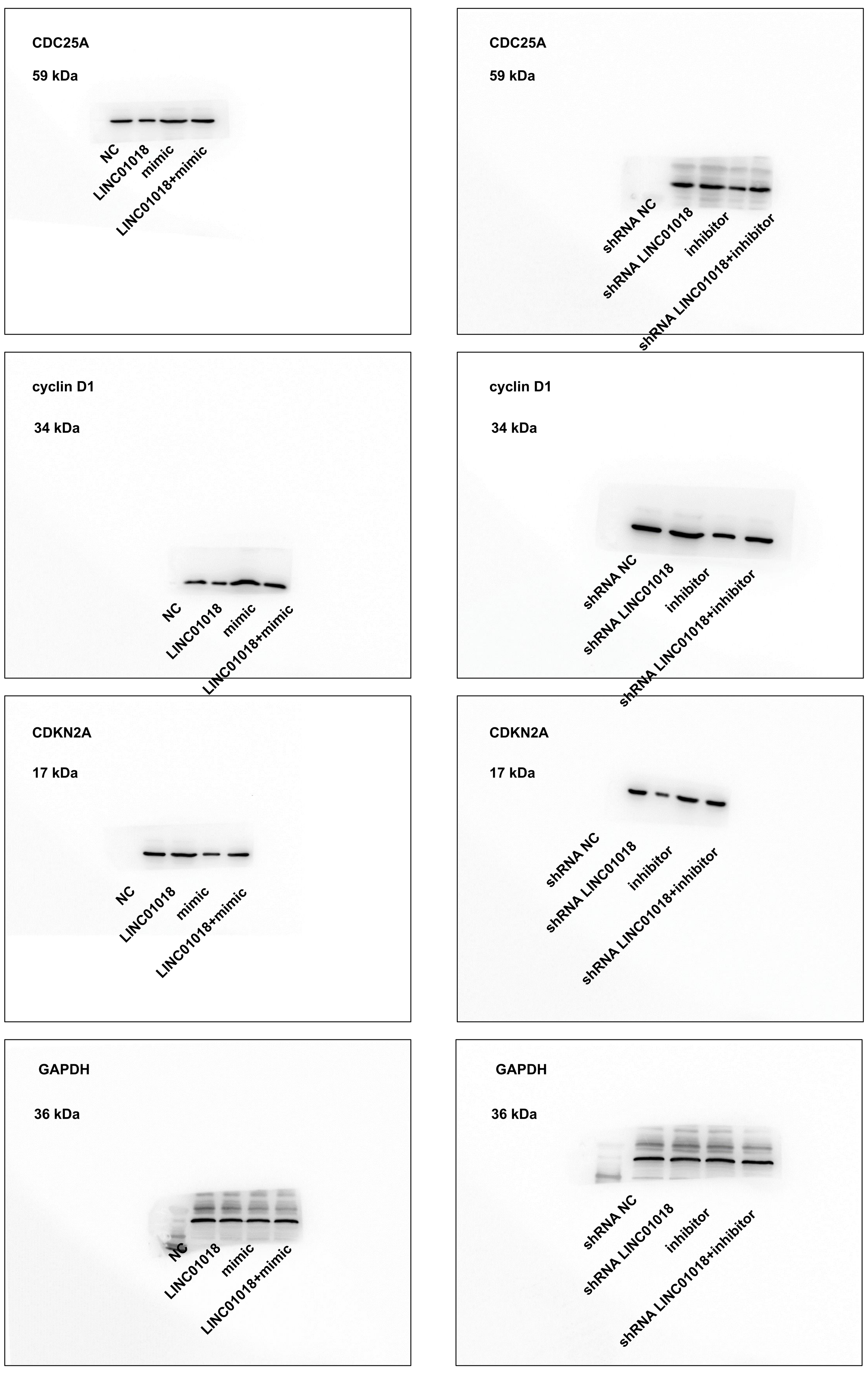

Full unedited gel/blot for Figure 12

LN-229 T98G

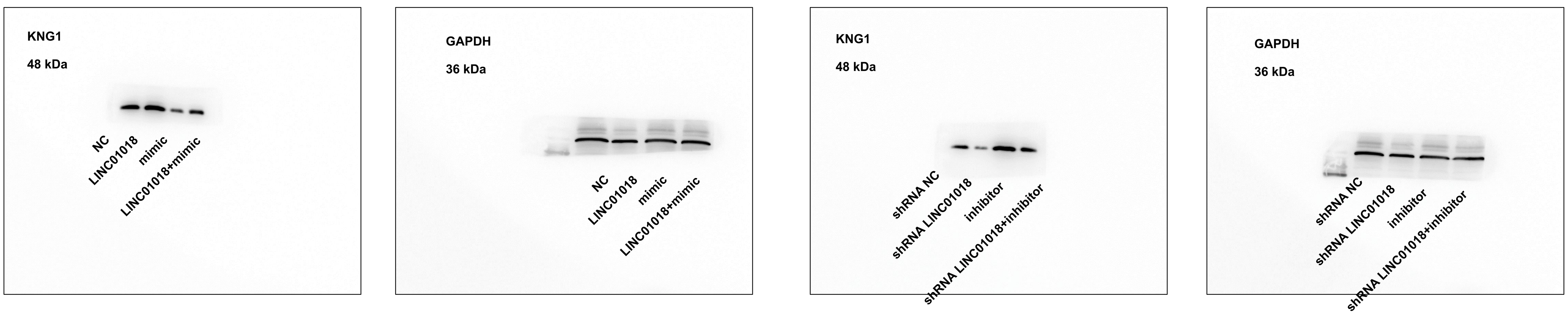

Supplement: Supplementary file 2 — AppendixS1 [file CNS-29-691-s002.pdf]
